# The cross-talk between PARylation and SUMOylation in C/EBPβ at K134 site participates in pathological cardiac hypertrophy

**DOI:** 10.7150/ijbs.65211

**Published:** 2022-01-01

**Authors:** Luping Wang, Panxia Wang, Suowen Xu, Zhuoming Li, Dayue Darrel Duan, Jiantao Ye, Jingyan Li, Yanqing Ding, Wenqing Zhang, Jing Lu, Peiqing Liu

**Affiliations:** 1 Department of Pharmacology and Toxicology, School of Pharmaceutical Sciences, Sun Yat-Sen University, Guangdong, China.; 2 Laboratory of Hematopathology & Drug Discovery, School of Medicine, South China University of Technology, Guangdong, China.; 3 Department of Endocrinology, The First Affiliated Hospital of USTC, Division of Life Sciences and Medicine, University of Science and Technology of China, Anhui, China.; 4 Center for Phenomics of Traditional Chinese Medicine/the Affiliated Hospital of Traditional Chinese Medicine, Southwest Medical University, Sichuan, China.; 5 National and Local United Engineering Lab of Druggability and New Drugs Evaluation, School of Pharmaceutical Sciences, Sun Yat-Sen University, Guangdong, China.; 6 School of Pharmacy, Jinan University, 601 Huangpu Avenue West, Guangdong, China.

**Keywords:** poly(ADP-ribosyl)ation, C/EBPβ, SUMOylation, cardiac hypertrophy

## Abstract

Poly(ADP-ribosyl)ation (PARylation) and SUMO modification (SUMOylation) are novel post-translational modifications (PTMs) mainly induced by PARP1 and SUMO1. Growing evidence has revealed that C/EBPβ plays multiple roles in biological processes and participates in cardiovascular diseases. However, the cross-talk between C/EBPβ PARylation and SUMOylation during cardiovascular diseases is unknown. This study aims to investigate the effects of C/EBPβ PTMs on cardiac hypertrophy and its underlying mechanism. Abdominal aortic constriction (AAC) and phenylephrine (PE) were conducted to induce cardiac hypertrophy. Intramyocardial delivery of recombinant adenovirus (Ad-PARP1) was taken to induce PARP1 overexpression. In this study, we found C/EBPβ participates in PARP1-induced cardiac hypertrophy. C/EBPβ K134 residue could be both PARylated and SUMOylated individually by PARP1 and SUMO1. Moreover, the accumulation of PARylation on C/EBPβ at K134 site exhibits downregulation of C/EBPβ SUMOylation at the same site. Importantly, C/EBPβ K134 site SUMOylation could decrease C/EBPβ protein stability and participates in PARP1-induced cardiac hypertrophy. Taken together, these findings highlight the importance of the cross-talk between C/EBPβ PTMs at K134 site in determining its protein level and function, suggesting that multi-target pharmacological strategies inhibiting PARP1 and activating C/EBPβ SUMOylation would be potential for treating pathological cardiac hypertrophy.

## Introduction

Pathological cardiac hypertrophy remains one of the main cardiovascular risk factors and represents poor prognosis associated with nearly all forms of heart failure 1. This pathological process is featured with enlargement of cardiomyocytes, pathological overexpression of fetal genes as well as cardiac contractile/diastole dysfunction. Although various hypertrophic risk factors such as hypertension, aortic stenosis, atherosclerosis, anterior wall myocardial infarction have been recognized 2, 3, the underlying molecular mechanisms are elusive and remain largely to be clarified. Post-translational modification (PTM) of proteins has gradually become a medical research hot spot, PTM is closely related to various cardiovascular diseases. PTM participates in protein kinase pathway response, mitochondrial oxidative stress, and regulation of downstream transcription factors, thereby affecting myocardial remodeling 4-6.

C/EBP (CCAAT/enhancer-binding proteins)-β, which is a transcription factor containing 3 main domains: a transactivation domain (N-terminal), a regulatory domain and a leucine zipper domain (C-terminal) 7. Previous study has shown that C/EBPβ inhibits the growth and proliferation of cardiomyocyte, and that the decrease in C/EBPβ is a pivotal signal during physiological cardiac hypertrophy 8. However, C/EBPβ has been found to play a different role in pathological cardiac hypertrophy. C/EBPβ protein and DNA binding activity are increased in cardiac hypertrophy, and downregulation of C/EBPβ attenuates phenylephrine (PE)-induced cardiomyocyte hypertrophy 9, 10. Myocardium-targeted C/EBPβ knockdown improves the cardiac function during cardiac hypertrophy 11. C/EBPβ could also be identified as a novel marker for metabolic disturbance during hypertrophy 12. Interruption of the epicardial C/EBP signaling pathway in adults can decrease neutrophil infiltration and ameliorate cardiac function 12. Notably, C/EBPβ protein could be greatly affected by numerous PTMs such as acetylation, phosphorylation, SUMOylation, ubiquitination and so on 13. Such PTMs in C/EBPβ were reported to regulate its protein stability, subcellular localization, DNA binding activity, or interaction with other transcription factors and coactivators 14, 15.

Poly(ADP-ribose) polymerase-1 (PARP1), the most important and best studied member in PARPs family, functions as DNA damage repair, transcriptional regulation, signal transduction and metabolic regulation. PARP1 transfers poly-ADP-ribose (PAR) groups from donor NAD^+^ molecules onto their target substrates, this PTM called poly(ADP-ribosyl)ation (PARylation) 16. PARylation performs many biological functions, including protein-protein or protein-DNA interaction, protein localization and stabilization, transcriptional activity of its substrates, and the regulation of other protein modifications 17. Previous studies demonstrated diverse transcription factors can be PARylated, such as p53, AP1, NFAT, Smads, FoxP3 and FoxO3 18-23. PARP1 is considered as a potential target for cardiovascular diseases, especially pathological cardiac hypertrophy. PARP1 knockdown mice showed amelioration of cardiac function in angiotensin II (Ang II) -triggered cardiac hypertrophy 24. Additionally, our previous studies also found that inhibitors of PARP1 protect against pathological hypertrophic responses induced via Ang II, PE or isoprenaline 25-28. Though some substrates are reported to be PARylated by PARP1, only a small minority of specific amino acid sites are identified. Whether or not the specific site of PARylation affects other post-translational modifications, and whether other modifications participate in PARP1-induced cardiac hypertrophy remain largely undetermined.

In this study, we have provided clear evidence that C/EBPβ PARylation and SUMOylation play opposite roles in cardiac hypertrophy. Mechanistically, the accumulation of PARylation of C/EBPβ at K134 site exhibits downregulation of C/EBPβ SUMOylation at the same site. Furthermore, C/EBPβ K134 site SUMOylation could decrease C/EBPβ protein stability and participates in PARP1-induced cardiac hypertrophy. Inhibition of C/EBPβ PARylation and activation of C/EBPβ SUMOylation represent a promising means to prevent cardiac hypertrophy.

## Materials and methods

### Animal experiment, anaesthesia and euthanasia

All animal experiments in this study were approved by Research Ethics Committee of Sun Yat-sen University and conformed to Guide for the Care and Use of Laboratory Animals (NIH Publication No. 85-23, revised 1996). SD rats (male, weighting 185-225g, SPF grade, Certification No. 44008500011930) were from the Experimental Animal Center of Sun Yat-sen University (Guangzhou, China). Sodium pentobarbital (40 mg/kg, Merck) was used for rat anesthetization before surgery, and then connected the noninvasive tracheal intubation to the ventilator under sterile conditions. The abdominal aorta above the kidney was exposed through a midline incision of the abdomen, and sutured twice with 6-0 silk sutures at the suprarenal level, tightly surrounding the aorta and a 22-gauge needle, the needle was then extracted to produce a 65-75% contraction. A similar procedure was performed in the Sham group, but without the aortic banding. 3-Aminobenzamide (3AB) (MedChem Express) was injected intraperitoneally (20 mg/kg, twice daily) starting the week after AAC surgery for 7 weeks. At the end of experiment, all rats were euthanized by injecting sodium pentobarbital (150 mg/kg, Merck) intraperitoneally for deep anaesthesia, followed by decapitation.

### Intramyocardial delivery of recombinant adenovirus

Recombinant adenovirus vectors harboring rat PARP1 cDNA (Ad-PARP1) and green fluorescent protein (Ad-GFP) were purchased from Genechem (Shanghai, China). Adenoviruses were amplified in 293A cell line, purified with a virus purification kit (Biomiga, San Diego, USA), then diluted to an appropriate titer and injected into the myocardium. Sodium pentobarbital (40 mg/kg, Merck) was used for rat anesthetization before surgery, and then connected the noninvasive tracheal intubation to the ventilator under sterile conditions. As described previously 23, the heart was exposed by thoracotomy through the left third to fourth intercostal space. Total 200 μL of Ad-PARP1 (10^10^ particles) or Ad-GFP (10^10^ particles) were injected into the left ventricle wall at 3-4 locations by using a sterile insulin syringe. Alpha-lipoic acid (ALA) was injected intraperitoneally (100 mg/kg) starting one week before the intramyocardial delivery of Ad-PARP1 and lasting for 3 weeks after surgery. After the operation, thoraces was closed and surgical incision was sutured.

### Echocardiography and histological analysis

Rats were anesthetized with 3% (v/v) isoflurane. Technos MPX ultrasound system (ESAOTE, Italy) was used to conduct the two-dimensionally guided M-mode echocardiography according to the methods described in our previous studies 28. Then, the M-mode recordings and parasternal short-axis parameters were measured. Basic cardiac function indexes, including ejection fraction (EF), fractional shortening (FS), left ventricular end-diastolic internal diameter at diastole (LVIDd), left ventricular end-systolic internal diameter (LVIDs), left ventricular end-diastolic volume (LV Vold), left ventricular end-systolic volume (LV Vols), left ventricular end-diastolic posterior wall thickness (LVPWd), left ventricular end-systolic posterior wall thickness (LVPWs), stroke volume (SV), left ventricular end-diastolic volume (LVEDV) and left ventricular systolic volume (LVSV) were measured. The animals were then sacrificed, and their hearts were quickly removed and weighed. The histological cross-sections of the heart tissues (5 μm thick) were fixed in 4% paraformaldehyde, embedded in paraffin blocks, and dyed with haematoxylins-eosin (HE), Masson and wheat germ agglutinin (WGA) staining for morphometry. The rest of the tissues were instantly frozen in liquid nitrogen and then preserved at -80 °C for further study.

### Cell Culture

Neonatal rat cardiomyocytes (NRCMs) were isolated from heart tissues of Sprague-Dawley rats (1- to 3-day-old) as described previously 29. Purified cardiomyocytes were seeded in plates using Dulbecco's modified Eagle Medium (Gibco, Grand Island, New York, USA) with 10% foetal bovine serum (Invitrogen, Carlsbad, CA, USA). To inhibit the growth cardiac fibroblasts, 0.1 mM 5-bromodeoxyuridine (Thermo Fisher Scientific, Waltham, MA, USA) was added, then the cells were cultured at 37 °C with 5% CO_2_. 24 h later, the cells were replaced with new medium and cultured for another 24 h before further studies.

### Measurement of cell surface area

NRCMs were fixed at room temperature using 4% paraformaldehyde for 30 min, and further treated with 0.3% TritonX-100 (Sangon Biotech) for 10 min. Cells were then washed with phosphate buffer solution (PBS) and incubated with 0.1% rhodamine-phalloidin (Invitrogen) for 1 h to visualize actin filaments. After washing with PBS, cells were further stained with DAPI (Cell Signaling Technology) and surface area was assessed using a high content screening system (Thermo Fisher Scientific). And cell surface area selected from random fields (50 for each group) were measured with the internally installed image analysis software.

### Quantitative real-time polymerase chain reaction (qRT-PCR)

Total RNA from NRCMs or rat heart tissues were extracted with TRIzol reagent (Invitrogen, USA). cDNA was then reversely transcribed using the Revert Aid First Strand cDNA Synthesis kit (Thermo Fisher Scientific). iCycler iQ system (iCycler, Bio-Rad) with SYBR-Green quantitative PCR kit (TOYOBO, Japan) was used to detect the mRNA expression levels of the target genes. Conditions of amplification reactions were as follows: 95 °C for 15 min, followed by 40 cycles of 95 °C for 30 s, 55 °C for 1 min, and 72 °C for 30 s. β-Actin was used to normalize as a housekeeping gene. Specific rat primers were synthesized by Sangon Biotech (listed in Supplementary Table S1).

### Western blot and co-immunoprecipitation (co-IP) analysis

The protein in NRCMs or rat heart tissues was extracted using RIPA lysis buffer (Beyotime, China). Concentrations of protein were conducted using a BCA Protein Assay Kit (Thermo Fisher Scientific, MA, USA). Equal protein amounts (30 μg) were loaded and separated by SDS-PAGE electrophoresis (8-12% SDS-PAGE gel). After transferring the proteins to polyvinylidene difluoride membranes (EMD Millipore Corporation, Billerica, MA, USA), the membranes were incubated with 5% defatted milk at room temperature for 1.5 h and then incubated with the indicated primary antibodies at 4 °C overnight and cultured with the secondary antibodies at room temperature for 1 h after that. Protein levels were detected with Image Quant LAS 4000 mini (Waukesha, WI, USA). The intensity was analysed using Image J software (Bio-Rad, Hercules, CA, USA).

The protein in NRCMs or rat heart tissues was extracted using IP lysis buffer (Beyotime, China). About 300 μg total proteins or 150 μg nuclear proteins were used for immunoprecipitation. The indicated antibodies or the corresponding IgG was added and incubated overnight at 4 °C. Then 30 μl of protein A/G plus agarose beads (Thermo Fisher, New York, USA) were added the next day and incubated for 4 hours at 4 °C. The beads were washed with IP washing buffer, and then boiled with 25 μl 2×loading buffer about 5 min.

The antibodies used in Western blot were as follows , anti-ANF antibody (diluted 1:200) was obtained from Santa Cruz Biotechnology, Inc. (Santa Cruz, CA, USA), anti-BNP antibody (diluted 1:1000) was purchased from Millipore (Billerica, Massachusetts, USA), anti-C/EBPβ antibody (diluted 1:1000) was obtained from Abcam (Cambridge, MA, USA), anti-PARP1 antibody (diluted 1:500) was purchased from Proteintech (Proteintech Group, Chicago, IL, USA), anti-SUMO1 (diluted 1:1000) were purchased from Cell Signaling Technology (Beverly, MA, USA). α-tubulin (mouse, diluted 1:5000), GAPDH (rabbit, diluted 1:1000) and c-MYC (rabbit, diluted 1:1000) antibodies were purchased from Sigma Aldrich (St. Louis, MO, USA). PAR antibody (mouse, diluted 1:500) was purchased from Trevigen (Gaithersburg, MD, USA).

For IP, anti-C/EBPβ (rabbit, diluted 1:10), anti-RFP antibody (mouse, diluted 1:10) were from Santa Cruz Biotechnology (Santa Cruz, CA, USA), and anti-PARP1 (rabbit, diluted 1:100), anti-HA (rabbit, diluted 1:20), anti-PAR (mouse, diluted 1:100) was purchased from Trevigen (Gaithersburg, MD, USA), anti-SUMO1 (rabbit, diluted 1:100) antibodies were purchased from Cell Signaling Technology (Beverly, MA, USA).

### Electrophoretic mobility shift assay (EMSA)

EMSA was conducted to analysis C/EBPβ DNA binding activity using the LightShift^TM^ chemiluminescent EMSA kit (Pierce, Rockford, IL, USA). Preparing a binding reaction system containing 1μg poly (dIdC), 0.05% Nonidet P-40, 5 mmol/L MgCl_2_, and 2.5% glycerol according to the reagent instructions. The nuclear proteins (5 μg) were incubated with the reaction system at room temperature for 10 min. Then biotin labeled C/EBPβ DNA probe was added and incubated at room temperature for 20 min. The reaction mixture was loaded and separated by 6% non-denaturing SDS-PAGE, transferred to nylon hybridization transfer membrane (Biodyne PLUS). After transferring to nylon hybridization transfer membrane (Biodyne PLUS), the membranes were then DNA cross-linked with UV (254 nm) for 10 min. The membranes were blocked at room temperature and shaken slowly for 1 h, then incubated with HRP-conjugated streptavidin antibodies for 15 min. Sequence of oligonucleotide used for EMSA was 5'-CGCCTTATTGCGCAATTGCCGC-3' (3'-biotinylated by Sangon, Shanghai).

### Plasmid transfection and RNA interference

The Flag-PARP1 (WT), Flag-PARP1 (E988K) and HA-C/EBPβ plasmids were purchased from Genechem (Shanghai, China), and the constructs were verified by DNA sequencing (Supplementary Figure S4). Two C/EBPβ mutants were produced by Generay (Shanghai, China), and the constructs were verified by DNA sequencing (Supplementary Figure S5 and S6). siRNA was synthesized by Genepharma (Shanghai, China). The sequences of siRNAs are listed in Supplementary Table S2. Lipofectamine 2000 reagent (Invitrogen, USA) was used to perform transient transfection in NRCMs according to the producer's protocol. qPCR and Western blot were conducted to confirm the silencing efficiency (Supplementary Figure S1).

### *In vitro* poly(ADP-ribosyl)ation (PARylation) assays

HEK293 cells expressed C-MYC-C/EBPβ recombinant protein were obtained from OriGene (Beijing, China), PARP1 recombinant protein, salmon sperm DNA and [^32^P] NAD^+^ were bought from Trevigen (Gaithersburg, MD, USA). Briefly, C-MYC-C/EBPβ were added in the mixture containing recombinant PARP1 protein, salmon sperm DNA and [^32^P] NAD^+^ with or without PARP inhibitor 3AB, PJ34 and AG14361 for 30 min at 37 °C. Western blot was conducted to detect the level of C-MYC-C/EBPβ PARylation.

### Immunofluorescence (IF) assay

NRCMs cultured on coverslips were fixed using 4% paraformaldehyde, washed with warm PBS, permeabilized with 0.3% Triton X-100 at room temperature for 10 min and subsequently incubated with 10% goat serum (BOSTER, Wuhan, China) at room temperature for 1 h. Afterwards, cells were incubated with primary antibody of PARP1 (diluted 1:50), PAR (diluted 1:50) and C/EBPβ (diluted 1:50) at 4 °C overnight, and further incubated with Alexa Fluor-labelled secondary antibody (diluted 1:200, Cell Signaling Technology, Danvers, MA, USA). Finally, the coverslips were mounted with DAPI (Cell Signaling Technology, USA). Confocal microscope (Zeiss, Germany) was used to detect the coverslips.

### Statistical Analysis

Data are shown as mean ± SD. Unpaired Student's t-tests was used to analyse the statistical significance between two groups, and analysis among multiple groups was conducted by one-way analysis of variance (ANOVA) with Bonferroni post-tests using GraphPad Prism 8.0 (San Diego, CA, USA). In all instances, a value of P < 0.05 was considered as statistically significant.

## Results

### PARP1 activity and C/EBPβ activity/expression are increased in models of cardiac hypertrophy

**Figure 1:**
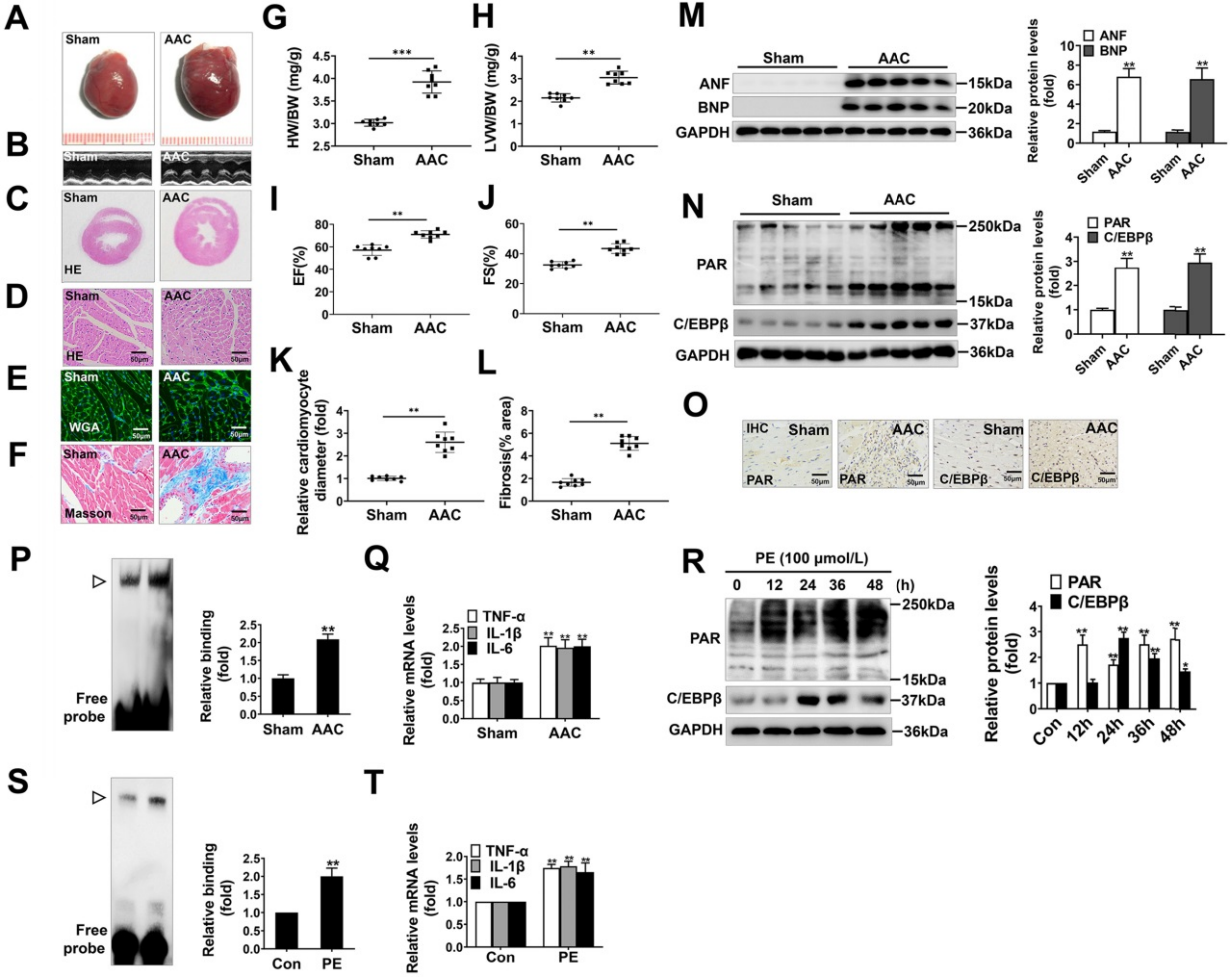
** PARP1 activity and C/EBPβ activity/expression are increased in models of cardiac hypertrophy.** Pressure overload was induced by AAC in rats, the hypertrophic changes in heart tissues were determined after 8 weeks of surgery. (**A**) Gross morphology of the hearts. (**B**) Representative images from echocardiography. (**C**-**F**) Representative hematoxylin-eosin (HE) stained cross sections of the left ventricle, wheat germ agglutinin (WGA) staining of cardiomyocytes and Masson staining of collagen deposition were shown, respectively. (**G, H**) HW/BW ratio and LVW/BW ratio were calculated. **P < 0.01 vs. Sham group, (n=8). (**I**, **J**) Echocardiographic parameters were measured. EF: ejection fraction; FS: fractional shortening. **P < 0.01 vs. Sham group, (n=8). (**K**, **L**) Relative cardiomyocyte diameter and Fibrosis (% area) were calculated. **P < 0.01 vs. Sham group, (n=8). (**M**) The protein levels of ANF and BNP were determined by Western blot. **P < 0.01 vs. Sham group, (n=8). (**N, O**) The relative PAR and C/EBPβ level were determined by IHC and Western blot. **P < 0.01 vs. Sham group, (n=4). (**P**) Electrophoretic mobility shift assay (EMSA) was performed using nuclear extract and a labeled double-stranded oligonucleotide probe corresponding to the C/EBPβ regulatory element. **▷**indicates C/EBPβ protein-DNA complex. **P < 0.01 vs. Sham group, (n=4). (**Q**) The mRNA expression of the target genes (TNF-α, IL-1β and IL-6) of C/EBPβ was determined by qRT-PCR. **P < 0.01 vs. Sham group, (n=4). (**R**) NRCMs were incubated with 100 μmol/L PE for the indicated durations. Western blot analysis was conducted to determine the protein levels of PAR and C/EBPβ. *P < 0.05 vs. Con group, **P < 0.01 vs. Con group, (n=3). (**S**) The DNA binding activity of C/EBPβ was analysed by EMSA. **▷**indicates C/EBPβ protein-DNA complex. *P < 0.05 vs. Con group, (n=3). (**T**) The mRNA expression of the target genes (TNF-α, IL-1β and IL-6) of C/EBPβ was determined by qRT-PCR. **P < 0.01 vs. PE group, (n=4).

AAC, widely known as a method to increase cardiac afterload, was typically employed to induce cardiac hypertrophy. In our study, the hearts of AAC-treated rats were significantly larger than those of the sham group (Figure 1A), and showed typical hypertrophic changes, like disorganized myocardial architecture, decreased intercellular space, and significant collagen deposition as revealed by HE staining, Masson staining, WGA staining and representative echocardiographic graphs (Figure 1B-1F, 1K, 1L). In addition, the heart-weight-to-body-weight ratio (HW/BW) (Figure 1G), left-ventricular-weight-to-body-weight ratio (LVW/BW) (Figure 1H) and echocardiographic parameters, such as EF and FS (Figure 1I, 1J) were markedly elevated. Furthermore, atrial natriuretic factor (ANF) and brain natriuretic polypeptide (BNP), which are the hypertrophic markers were also remarkably increased in AAC rat hearts (Figure 1M). These results demonstrate that cardiac hypertrophy was successfully induced by AAC.

Additionally, we found the protein level of C/EBPβ and the PARylation level of total proteins were augmented in AAC rat hearts, as implied by results of Western blot (Figure 1N) and immunohistochemical (IHC) (Figure 1O). The C/EBPβ DNA binding activity was also enhanced following AAC treatment (Figure 1P). Due to its diverse target genes, C/EBPβ participates in the pathological process particularly through upregulating tumor necrosis factor-α (TNF-α), interleukin-1β (IL-1β) and interleukin-6 (IL-6) 30-32, which are pivotal factors mediating cardiac inflammatory responses during hypertrophy. The mRNA expression of C/EBPβ-target genes (TNF-α, IL-1β and IL-6) were notably increased by AAC treatment (Figure 1Q). Accordingly, the expression and activity of C/EBPβ and PARP1 were further investigated in PE-treated NRCMs. As shown in Figure 1R, the cellular PARylation and C/EBPβ protein levels were elevated by PE treatment in a time-dependent manner. Additionally, the DNA binding activity of C/EBPβ (Figure 1S) and its target genes (TNF-α, IL-1β and IL-6) (Figure 1T) were also enhanced following PE treatment. These results demonstrate PARP1 activity and C/EBPβ activity/expression are increased in models of cardiac hypertrophy.

### PARP1 induces cardiac hypertrophy and increases C/EBPβ protein level *in vivo*

**Figure 2:**
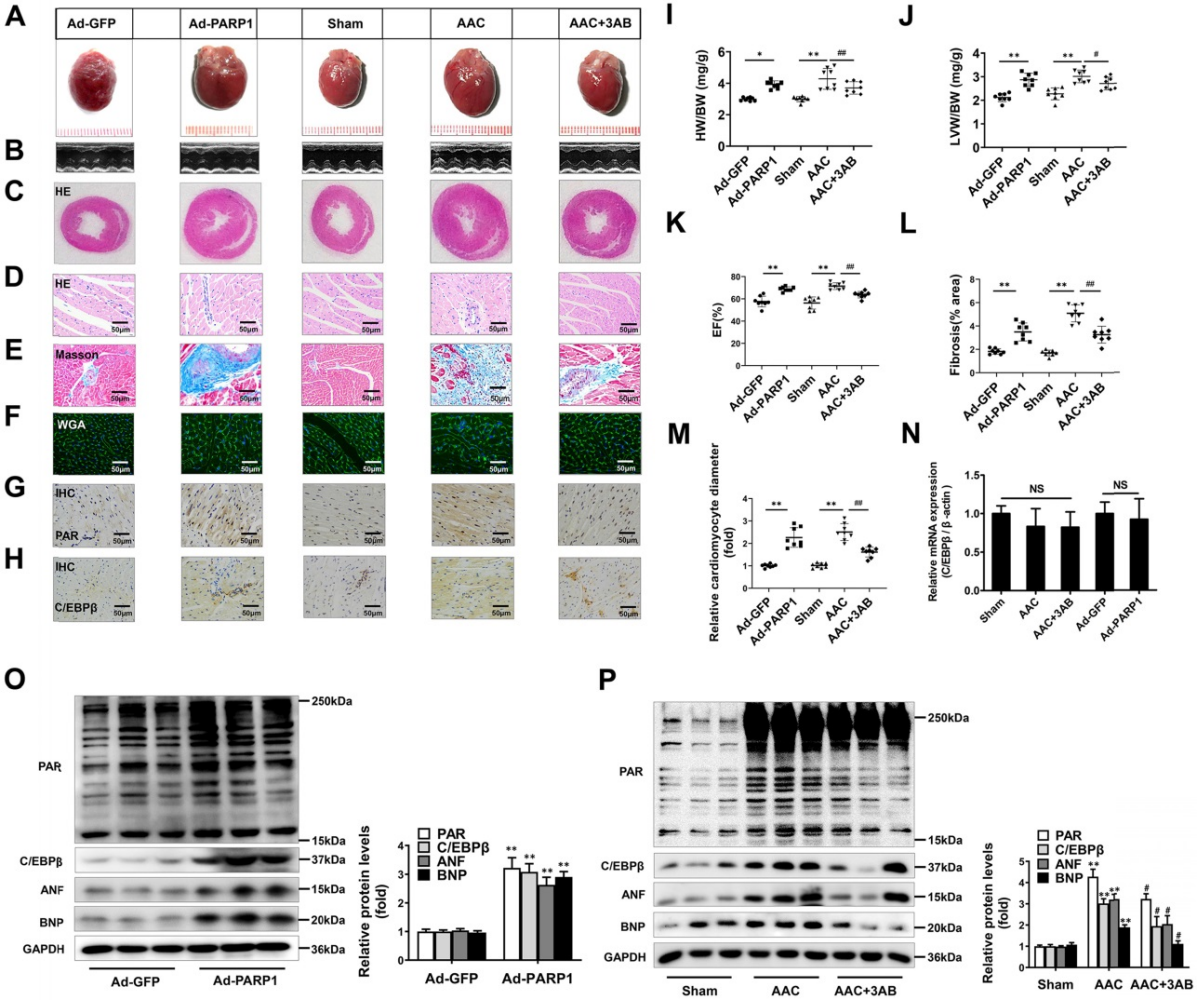
** PARP1 induces cardiac hypertrophy and increases C/EBPβ protein level *in vivo*.** Rats were submitted to the intracardiac injection of adenovirus encoding PARP1 (Ad-PARP1, 10^10^ particles), the control animals received adenovirus encoding green fluorescent protein (Ad-GFP, 10^10^ particles). Additionally, pressure overload was induced by abdominal aortic constriction (AAC) in rats, PARP1 inhibitor 3AB was intraperitoneally injected (20 mg/kg, twice daily) starting the week after AAC surgery for 7 weeks. (**A-F**) Pathological changes in the heart tissues were measured by HE staining, Masson staining, WGA staining and echocardiography, the representative graphs were shown, respectively. (**G**, **H**) IHC were conducted to detect the levels of PAR and C/EBPβ. (**I, J**) HW/BW ratio and LVW/BW ratio were calculated. **P < 0.01 vs. Sham group, ^#^P < 0.05 *vs*. the AAC group, (n=8). (**K**) Echocardiographic parameters were measured. EF: ejection fraction. **P < 0.01 vs. Sham group, ^##^P < 0.01 *vs*. AAC group, (n=8). (**L**, **M**) Relative fibrosis (% area) and cardiomyocyte diameter were calculated. **P < 0.01 vs. Sham group, ^##^P < 0.01 *vs*. AAC group, (n=8). (**N**) The mRNA expression of C/EBPβ was determined by qRT-PCR. (n=5). (**O**, **P**) The protein levels of PAR, C/EBPβ, ANF and BNP were determined by Western blot. **P < 0.01vs. Ad-GFP or Sham group,^ #^P < 0.05 vs. AAC group, (n=8).

PARP1 has been regarded as a promising target for cardiovascular disorders, especially pathological cardiac hypertrophy 33, 34. Considering that C/EBPβ activity/expression and PARP1 activity (PARylation) were both augmented in cardiac hypertrophy, we further explored whether the change of C/EBPβ was associated with PARP1. PARP1 adenovirus vector or a well-confirmed PARP1 inhibitor 3-aminobenzamide (3AB) was respectively used in SD rats. According to previous reports 23, PARP1 was overexpressed in cardiomyocytes by intracardiac multi-point delivery of Ad-PARP1. We found that PARP1 overexpression caused significant cardiac hypertrophy and fibrosis, as evaluated by gross morphologic examination (Figure 2A), echocardiography (Figure 2B, 2K and Supplementary Table S3), HE staining (Figure 2C, 2D), Masson staining (Figure 2E, 2L), WGA staining (Figure 2F, 2M), the increased ratios of HW/BW (Figure 2I) and LVW/BW (Figure 2J), as well as the increased protein levels of ANF and BNP (Figure 2O). Additionally, rats were injected with 3AB intraperitoneally (20 mg/kg, twice daily) starting the week after AAC surgery for 7 weeks. AAC pressure overload remarkably induced cardiac hypertrophy, while these effects were attenuated by 3AB treatment (Figure 2A-2F, 2I-2M, 2P and Supplementary Table S4). These results indicated that PARP1 could induce cardiac hypertrophy.

Ad-PARP1 in rat hearts induced increased protein level of C/EBPβ and the PARylation levels of total proteins, as implied by results of IHC (Figure 2G, 2H) and Western blot (Figure 2O). Contrarily, PARP1 inhibitor 3AB decreased the protein level of C/EBPβ and the PARylation levels of total proteins (Figure 2G, 2H, 2P). Intriguingly, the mRNA expression of C/EBPβ was not affected either by Ad-PARP1 or AAC treatment (with or without 3AB) (Figure 2N). Taken together, the results above suggest that PARP1 could induce cardiac hypertrophy and positively regulate C/EBPβ protein level without affecting its transcription level, which imply C/EBPβ may participate in PARP1-induced cardiac hypertrophy.

### C/EBPβ participates in PARP1-induced cardiac hypertrophy *in vitro* and *in vivo*

**Figure 3:**
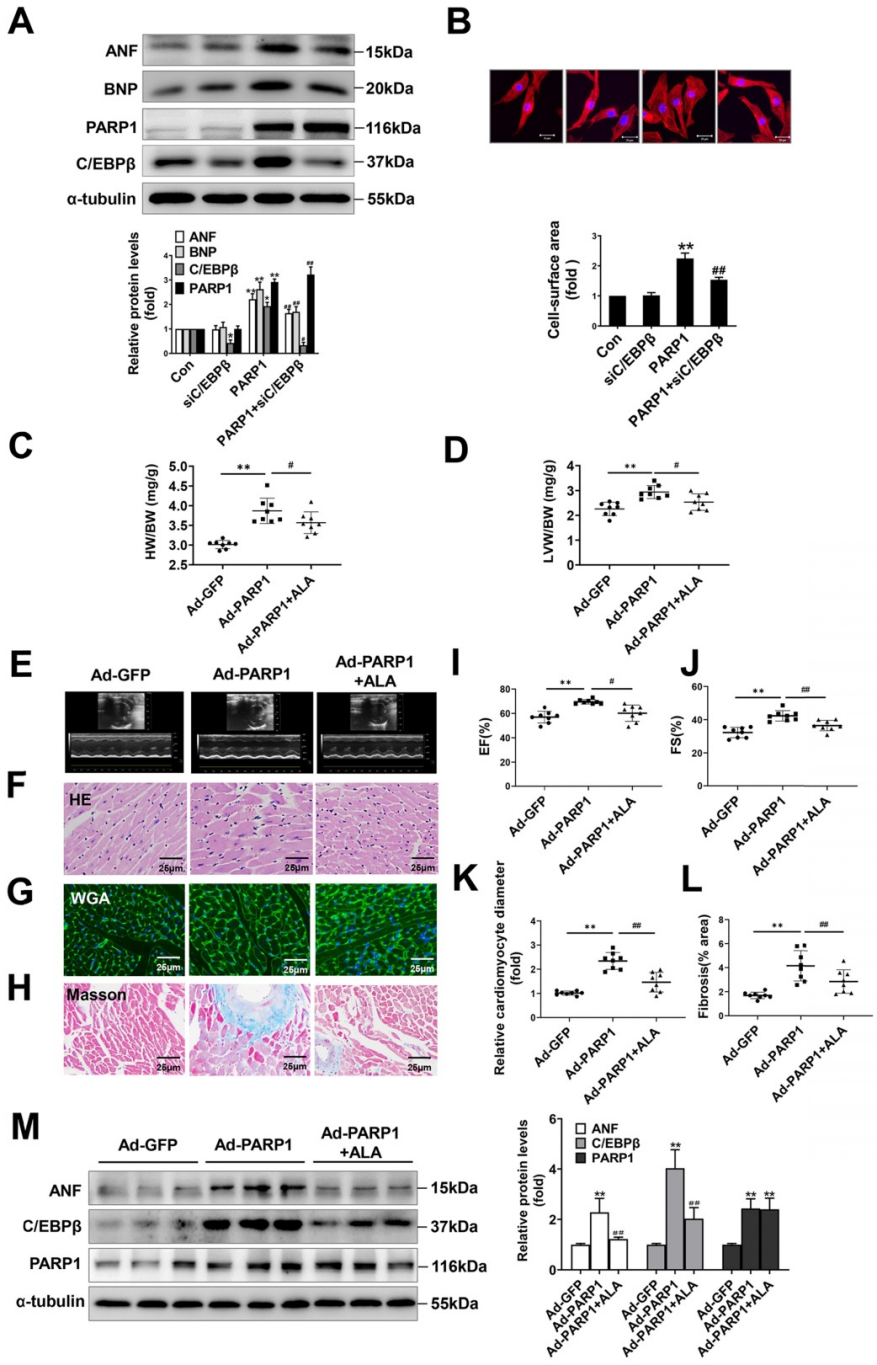
** C/EBPβ participates in PARP1-induced cardiac hypertrophy *in vitro* and* in vivo*.** C/EBPβ was knocked down by RNA interference in NRCMs infected with Ad-PARP1. (**A**) the protein levels of ANF and BNP were determined by western blot. *P < 0.05, **P < 0.01 vs. Con group, ^#^*P* < 0.05, ^##^*P* < 0.01vs. Ad-PARP1 group, (n=3). (**B**) The cell surface area was measured by staining with rhodamine-phalloidin. **P < 0.01 vs. Con group, ^##^P < 0.01 vs. Ad-PARP1 group, (n=3). Rats were submitted to the intracardiac injection of adenovirus encoding PARP1 (Ad-PARP1, 10^10^ particles), the control animals received adenovirus encoding green fluorescent protein (Ad-GFP, 10^10^ particles). Alpha-lipoic acid (ALA) was injected intraperitoneally (100 mg/kg) starting one week before the intramyocardial delivery of Ad-PARP1 and lasting for 3 weeks after surgery. (**C, D**) HW/BW ratio and LVW/BW ratio were calculated. **P < 0.01 vs. Ad-GFP group, ^#^P < 0.05 *vs*. Ad-PARP1 group, (n=8). (**E-H**) Pathological changes in the heart tissues were measured by echocardiography, HE staining, WGA staining and Masson staining, the representative graphs were shown, respectively. (**I**, **J**) Echocardiographic parameters were measured. EF: ejection fraction; FS: fractional shortening. **P < 0.01 vs. Ad-GFP group, ^#^P < 0.05 *vs*. Ad-PARP1 group, (n=8). (**K**, **L**) Relative cardiomyocyte diameter and Fibrosis (% area) were calculated. **P < 0.01 vs. Ad-GFP group, ^##^P < 0.01 *vs*. Ad-PARP1 group, (n=8). (**M**) The protein levels of ANF, C/EBPβ and PARP1 were determined by Western blot. **P < 0.01 vs. Ad-GFP group, ^##^P < 0.01 *vs*. Ad-PARP1 group, (n=4).

To ascertain the role of PARP1 and C/EBPβ in PE-induced cardiac hypertrophy, endogenous PARP1 and C/EBPβ were knocked down using the appropriate siRNA in NRCMs (Supplementary Table S2 and Supplementary Figure S1A-S1D). siPARP1 transfection or 3AB treatment attenuated PE-induced enlargement of cell surface area (Supplementary Figure S2A) and increases of ANF and BNP mRNA expressions (Supplementary Figure S2B), which confirms that PARP1 inhibition or deficiency could improve cardiomyocyte hypertrophy. Additionally, C/EBPβ overexpression led to remarkable enlargement of cell surface area (Supplementary Figure S2C) and elevation of expressions of hypertrophic marker genes (Supplementary Figure S2D). Conversely, C/EBPβ knockdown attenuated hypertrophic responses triggered by PE, as confirmed by the changes of cell surface area (Supplementary Figure S2E) and expressions of hypertrophic marker genes (Supplementary Figure S2F). Notably, knockdown of C/EBPβ significantly attenuated cardiomyocyte hypertrophy induced by PARP1 overexpression in NRCMs (Figure 3A, 3B). Alpha-lipoic acid (ALA) is a naturally occurring compound which has been reported to attenuate cardiac hypertrophy via inhibition of C/EBPβ activation 9. The treatment of ALA significantly attenuated PARP1 induced hypertrophic responses, as shown by ratios of HW/BW and LVW/BW (revised Figure 3C, 3D), representative echocardiographic graphs and parameters (revised Figure 3E, 3I, 3J and Supplementary Table S3), HE and WGA staining (revised Figure 3F, 3G, 3K), and Masson staining (revised Figure 3H, 3L). Furthermore, the hypertrophic marker ANF was also remarkably decreased (revised Figure 3M). Which is the first time to demonstrate C/EBPβ is at least partially participate in PARP1-initiated cardiac hypertrophy.

**Figure 4:**
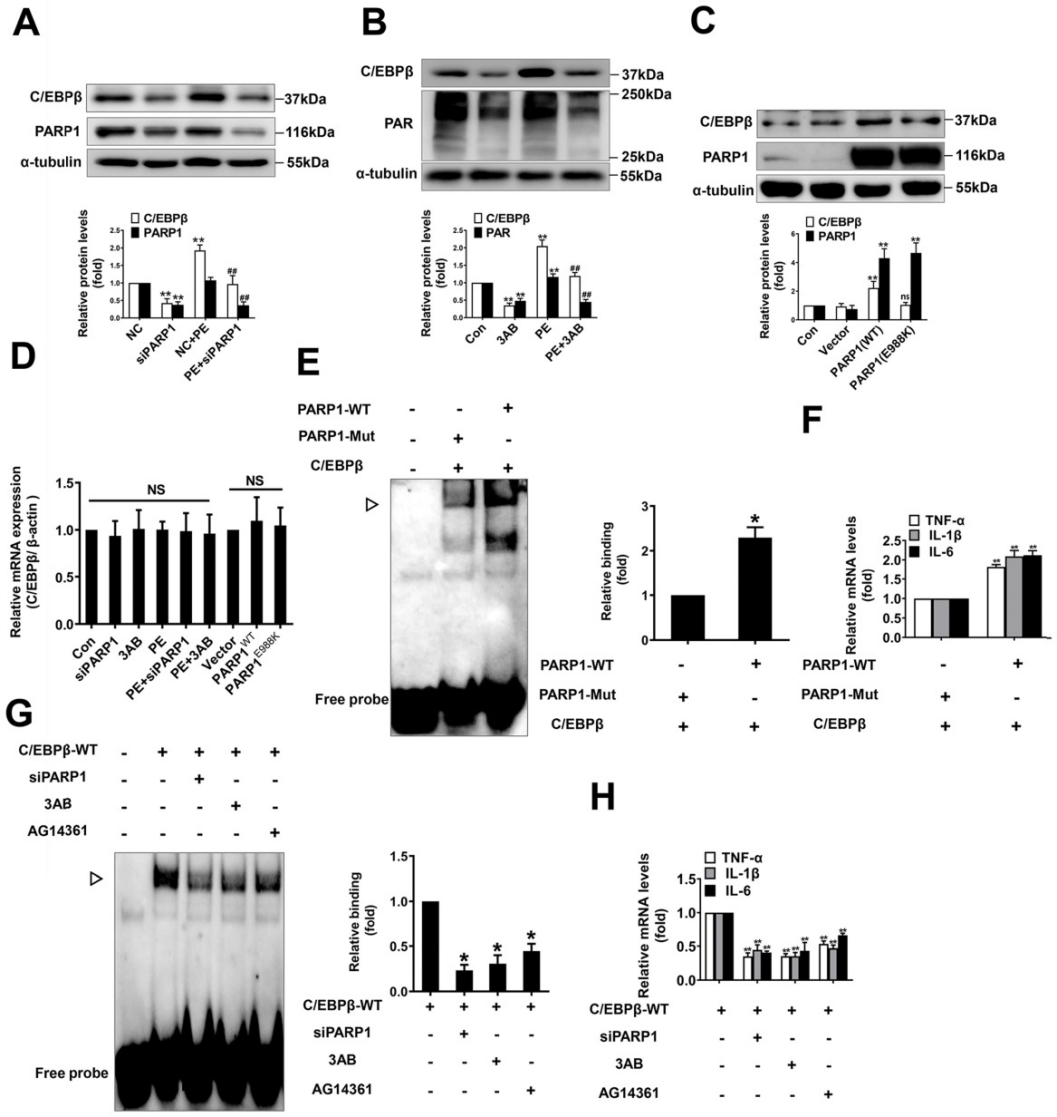
** PARP1 positively regulate C/EBPβ protein expression and DNA binding activity.** (**A**) NRCMs were transfected with siPARP1 followed by 100 μmol/L PE for 24 h. Western blot was conducted to determine the protein expression of C/EBPβ. **P < 0.01 vs. NC group, ^##^*P* < 0.01 vs. PE group, (n=3). (**B**) NRCMs were incubated with PARP1 inhibitor 3-aminobenzamide (3AB) (20 μmol/L) followed by 100 μmol/L PE for 24 h. Western blot was conducted to determine the protein expression of C/EBPβ. **P < 0.01 vs. Con group, ^##^*P* < 0.01 vs. PE group, (n=3). (**C**) NRCMs were transfected with wild-type PARP1 or mutant E988K for 48 h. The protein levels of C/EBPβ were determined by Western blot. **P < 0.05 vs. Con group, (n=3). (**D**) The mRNA expression of C/EBPβ was determined by qRT-PCR. (n=5). (**E**) PARP1-WT and PARP1-E988K (mutant) plasmids were expressed in NRCMs. EMSA was then conducted to analysis C/EBPβ DNA banding activity. *P < 0.05 vs. PARP1-Mut group, (n=3). **▷** indicates C/EBPβ protein-DNA complex. (**F**) The mRNA expression of the target genes (TNF-α, IL-1β and IL-6) of C/EBPβ was determined by qRT-PCR. ^##^P < 0.01 vs. PARP1-Mut group, (n=3). (**G**) NRCMs were transfected with the vector for HA-WT C/EBPβ followed by transfection with PARP1 siRNA or incubation with 3AB (20 μmol/L) or AG14361 (1 μmol/L). EMSA was then conducted to analysis C/EBPβ DNA banding activity. *P < 0.05 vs. Con group, (n=3). **▷** indicates C/EBPβ protein-DNA complex. (**H**) The mRNA expression of the target genes (TNF-α, IL-1β and IL-6) of C/EBPβ was determined by qRT-PCR. **P < 0.01 vs. Con group, (n=3).

In addition, we studied whether C/EBPβ are regulated by PARP1 in NRCMs. Firstly, knockdown or inhibition of PARP1 reversed PE-induced increase of C/EBPβ protein level (Figure 4A, 4B), which are in line with the *in vivo* results. It is well demonstrated glutamic acid residue at 988 in PARP1 is mainly responsible for its enzymatic activity, PARP1-E988K (the glutamic acid residue at 988 was replaced with a lysine residue) has no catalytic activity 35. As shown in Figure 4C, the protein level of C/EBPβ was prominently increased in cardiomyocytes overexpressing wild-type PARP1, however, it was not altered in those cells transfected with the mutant PARP1-E988K (Figure 4C). Interestingly, the mRNA expression of C/EBPβ was also not changed either by PARP1 overexpression or silencing/inhibition (Figure 4D), suggesting that the regulation of PARP1 on C/EBPβ protein level may be independent of its transcription. Finally, we investigated whether PARP1 regulates C/EBPβ DNA binding activity and its target genes expression. Overexpression of wild-type PARP1 but not the PARP1-E988K mutant enhanced the DNA binding activity of C/EBPβ (Figure 4E) and the target genes (TNF-α, IL-1β and IL-6) expression (Figure 4F). While knockdown or inhibition of PARP1 decreased C/EBPβ DNA binding activity (Figure 4G) and the target genes (TNF-α, IL-1β and IL-6) expression (Figure 4H). In summary, these results imply that C/EBPβ participates in PARP1-initiated cardiac hypertrophy, PARP1 positively regulate C/EBPβ protein level and DNA binding activity without affecting its mRNA level, suggesting that this regulation is not at the transcriptional level, but is probably attributed to the stability or degradation of C/EBPβ protein.

### PARP1 interacts with C/EBPβ and PARylates C/EBPβ

**Figure 5:**
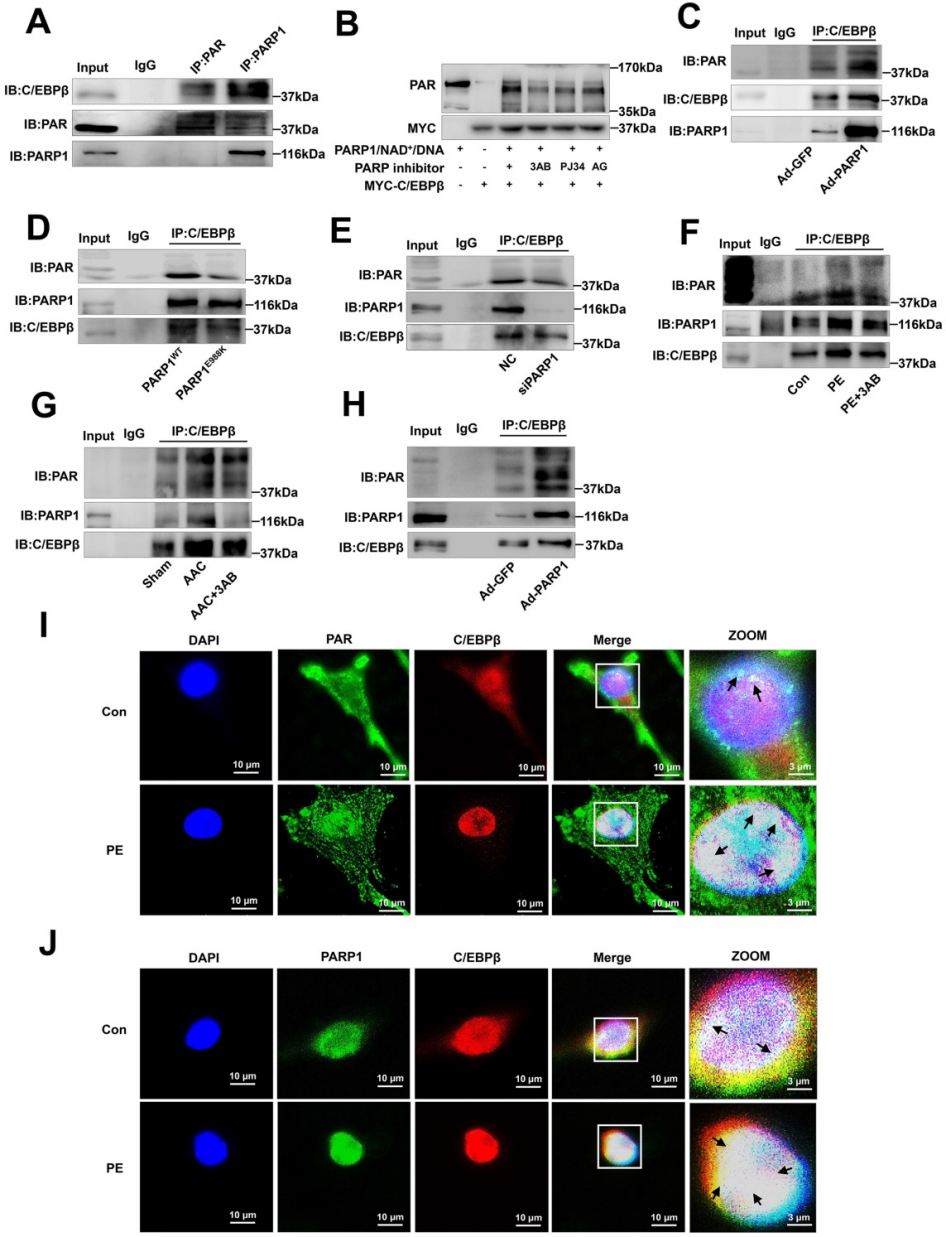
** PARP1 interacts with C/EBPβ and PARylates C/EBPβ.** (**A**) The nuclear fraction of NRCMs was precipitated by PARP1 and PAR antibody, followed by subjection to IP assays. (**B**) HEK293 cells expressed MYC-C/EBPβ protein were incubated within the PARylation reaction buffer with PARP1 protein (with or without PARP1 inhibitor: 3AB, PJ34 and AG14361(AG)), salmon sperm DNA and NAD^+^ for 30 minutes at 37 °C. PARylation of MYC-C/EBPβ was detected by anti-PAR antibody by Western blot. (**C**,** D**) NRCMs were transfected with WT PARP1 or mutant E988K and were precipitated by anti-C/EBPβ antibody for PARP1 and PAR detection in co-IP assays. (**E**) NRCMs were transfected with siPARP1, immunoprecipitated with anti-C/EBPβ antibody and subsequently subjected to immunoblotting analysis. (**F**) NRCMs were incubated with 3AB (20 μmol/L) followed by incubation with PE (100 μmol/L for 24 h), and then precipitated by anti-C/EBPβ antibody for PARP1 and PAR detection in IP assays. (**G**) PARP1 inhibitor 3AB was intraperitoneally injected (20 mg/kg, twice daily) starting the week after AAC surgery for 7 weeks. The rat heart tissue was immunoprecipitated with anti-C/EBPβ antibody and subsequently subjected to Western blot analysis. (**H**) SD rats were submitted to the intracardiac injection of adenovirus encoding PARP1 (Ad-PARP1, 10^10^ particles), the control animals received green fluorescent protein (Ad-GFP, 10^10^ particles). The rat heart tissue was immunoprecipitated with anti-C/EBPβ antibody and subsequently subjected to Western blot analysis. (**I**) The intracellular co-localization of PAR and C/EBPβ were confirmed in NRCMs, by confocal immunofluorescence microscopy with or without PE treatment. (**J**) The intracellular co-localization of PARP1 and C/EBPβ were confirmed in NRCMs, by confocal immunofluorescence microscopy with or without PE treatment. Representative images of three independent experiments are shown. n=3.

To explore the mechanisms by which C/EBPβ participates in PARP1-induced cardiac hypertrophy, we hypothesized that PARP1 might interact with C/EBPβ and PARylate it. According to IP results, C/EBPβ was precipitated by PARP1 and PAR antibody in the nuclear fraction of NRCMs (Figure 5A). We also performed *in vitro* PARylation assay via a reaction system containing purified MYC-C/EBPβ protein and recombinant PARP1 protein, as shown in Figure 5B, MYC-C/EBPβ protein could be PARylated and the level of PARylation reduced when given PARP1 inhibitors. Additionally, the gain- and loss-of-function of PARP1 were applied to test the interactions of the two proteins and the PARylation level of C/EBPβ. In NRCMs infected with Ad-PARP1, the interactions of PARP1 and C/EBPβ were enhanced, and the PARylation level of C/EBPβ was increased as well (Figure 5C). By contrast, when transfected with PARP1(E988K) mutant plasmid, the PARP1-C/EBPβ interaction was weakened and the level of C/EBPβ PARylation was restrained (Figure 5D). The similar results were also observed when transfected with PARP1 siRNA (Figure 5E) or given PARP1 inhibitor 3AB (Figure 5F) in NRCMs. In the heart tissues of AAC rats, both the C/EBPβ-PARP1 interaction and C/EBPβ PARylation were increased, and PARP1 inhibitor 3AB alleviated the interactions of the two proteins and C/EBPβ PARylation (Figure 5G). While in the hearts of rats injected with Ad-PARP1 remarkably increased C/EBPβ PARylation level (Figure 5H). Additionally, the intracellular co-localization of PAR and C/EBPβ or that of PARP1 and C/EBPβ in NRCMs were both confirmed by immunofluorescence (IF). As demonstrated in Figure 5I, PAR was mainly localized in the peri-nuclear at physiological status, while when treated with PE, PAR was significantly increased especially in the nucleus, and the co-localization of PAR and C/EBPβ was significantly increased by PE as well. Similarly, the co-localization of PARP1 and C/EBPβ was observed in NRCMs, and PE treatment also increased the co-localization of PARP1 and C/EBPβ (Figure 5J). Taken together, these above results confirm the fact that PARP1 interacts with C/EBPβ and PARylates it.

### SUMOylation of C/EBPβ at K134 site regulates C/EBPβ protein stability and prevents cardiac hypertrophy

**Figure 6:**
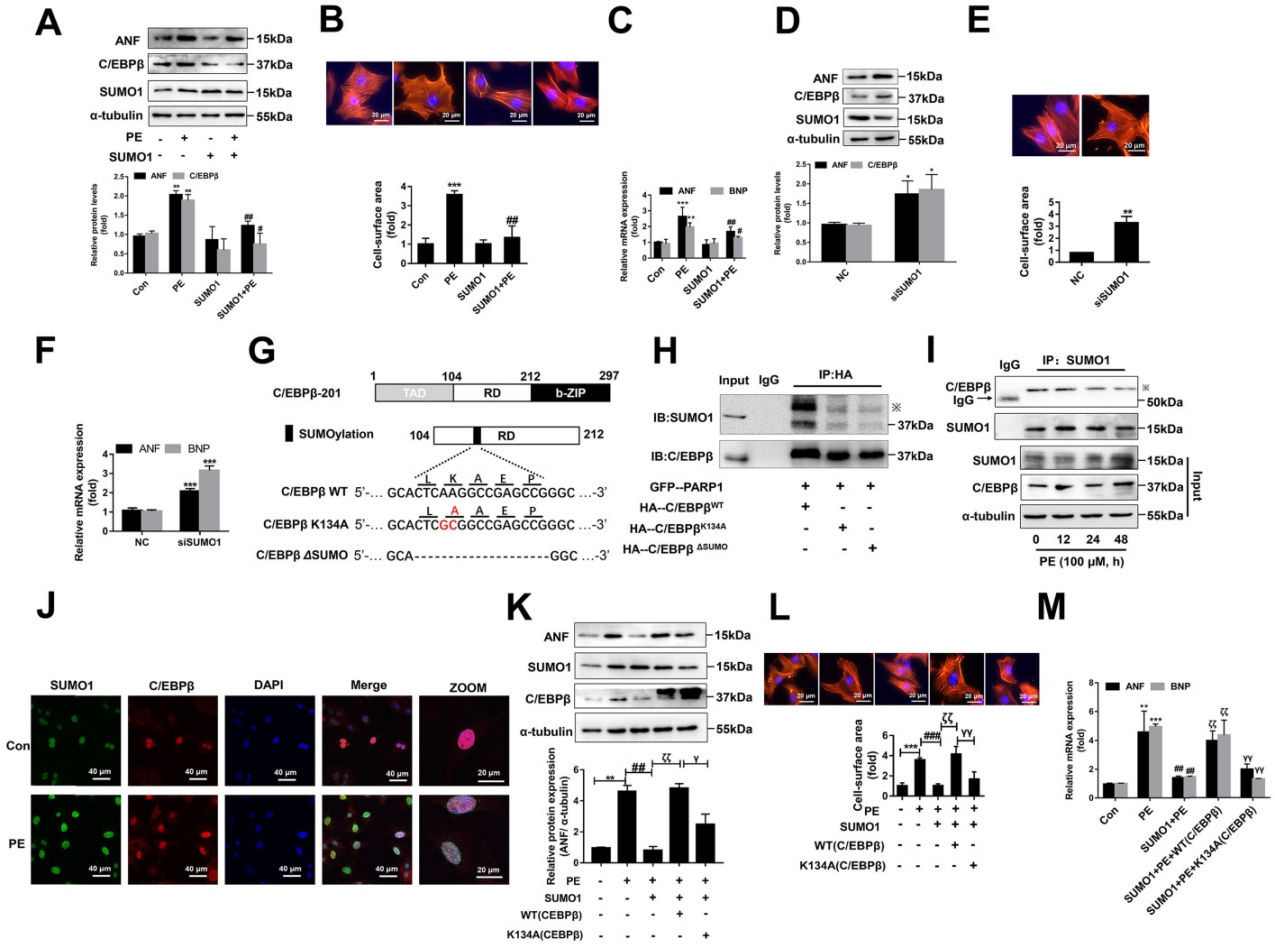
** SUMOylation of C/EBPβ at K134 site regulates C/EBPβ protein stability and prevents cardiac hypertrophy.** (**A**) NRCMs were transfected with RFP-SUMO1 with or without PE treatment (100 μmol/L for 24 h), the protein levels of ANF and C/EBPβ were determined by western blot. **P < 0.01 vs. Con group, ^#^*P* < 0.05, ^##^*P* < 0.01 vs. PE group, (n=3). (**B**) Cell surface area was measured by rhodamine staining. *P < 0.05 vs. Con group,^ ##^*P* < 0.01 vs. PE group, (n=3). (**C**) The mRNA levels of ANF and BNP were determined by qRT-PCR. **P < 0.01, ***P < 0.001 vs. Con group,^ #^*P* < 0.05, ^##^*P* < 0.01 vs. PE group, (n=3). (**D**) NRCMs were transfected with SUMO1 siRNA, the protein levels of C/EBPβ and ANF were determined by western blot. *P < 0.05 vs. NC group, (n=3). (**E**) Cell surface area was measured by rhodamine staining. **P < 0.01 vs. NC group, (n=3). (**F**) The mRNA levels of ANF and BNP were determined by qRT-PCR. ***P < 0.001 vs. Con group, (n=3). (**G**) The diagram of C/EBPβ protein domain, and the two mutant/truncated plasmids of C/EBPβ. (**H**) GFP-PARP1, HA-C/EBPβ^WT^, HA-C/EBPβ^K134A^ and HA-C/EBPβ^ΔSUMO^ were expressed in NRCMs, nuclear extracts were subjected to IP with anti-HA antibody followed by Western blot with anti-SUMO1, anti-C/EBPβ antibody. ※ indicates C/EBPβ-SUMO. n=3. (**I**) NRCMs were incubated with 100 μmol/L PE for the indicated durations, immunoprecipitated with anti-C/EBPβ antibody and subsequently subjected to Western blot analysis. n=3. (**J**) The intracellular co-localization of SUMO1 and C/EBPβ were confirmed in NRCMs, by confocal immunofluorescence microscopy with or without PE treatment (100 μmol/L for 24 h). n=3. (**K**) NRCMs were transfected with the expression vectors for RFP-SUMO1, HA-WT C/EBPβ and HA-K134A C/EBPβ as indicated followed by PE treatment (100 μmol/L for 24 h). The protein level of ANF was determined by western blot. **P < 0.01 vs. Con group,^ ##^*P* < 0.01 vs. PE group, ^ζζ^*P* < 0.01 vs. PE+SUMO1 group, ^γ^*P* < 0.05 vs. PE+SUMO1+WT C/EBPβ group. (n=3). (**L**) Cell surface area was measured by rhodamine staining. ***P < 0.001 vs. Con group,^ ###^*P* < 0.001 vs. PE group, ^ζζ^*P* < 0.01 vs. PE+SUMO1 group, ^γγ^*P* <0.01 vs. PE+SUMO1+WT C/EBPβ group. (n=3). (**M**) The mRNA levels of ANF and BNP were determined by qRT-PCR. *P < 0.05 vs. the Control group, **P < 0.01, ***P < 0.001 vs. Con group,^ ##^*P* < 0.01 vs. PE group, ^ζζ^*P* < 0.01 vs. PE+SUMO1 group, ^γγ^*P* < 0.01 vs. PE+SUMO1+WT C/EBPβ group. (n=3).

C/EBPβ and the other family members have a conserved regulatory domain (RD) motif which is subjected to SUMOylation 36. SUMOylation of C/EBPβ was reported dramatically enhances its ubiquitination and protein destabilization 37. From above results, we have demonstrated that C/EBPβ PARylation positively regulate its protein level without affecting its mRNA expression, thus we speculated there may be a crosstalk between C/EBPβ PARylation and SUMOylation in determining its protein stability. Firstly, we studied whether SUMO1 regulates C/EBPβ protein level and participates in cardiac hypertrophy. Overexpression of SUMO1 could reverse PE-induced increase of C/EBPβ protein level (Figure 5A) and cardiac hypertrophy (Figure 6A-6C). Conversely, SUMO1 knockdown in NRCMs decreased C/EBPβ SUMOylation (Supplementary Figure S3A), while increased C/EBPβ protein level (Figure 6D), the hypertrophic responses were also induced by SUMO1 knockdown in NRCMs (Figure 6D-6F).

Considering the result that SUMO1 negatively regulate C/EBPβ protein level, we speculated SUMO1 interacts with C/EBPβ and leads the instability of its protein. Previous studies have reported that on the RD motif (LKAEP) in rat C/EBPβ, 134 lysine residue (K134) is considered the likely site for attachment of SUMO1 15, 37, 38. C/EBPβ interacts with PIAS1 (a SUMO E3 ligase) and is SUMOylated at Lys133 (K134 in rat) site, inducing ubiquitination and degradation of C/EBPβ protein 39. Next, we investigated whether C/EBPβ RD motive (LKAEP) or K134 site is responsible for the attachment of SUMO1.

To test this hypothesis, we generated two mutant/truncated plasmids of C/EBPβ (Figure 6G): (1) single mutant with alanine substitution at K134 (K134A); (2) truncation of conserved motif (LKAEP) named ΔSUMO. The SUMOylation of C/EBPβ in NRCMs transfected with these mutants were tested by IP assays. As shown in Figure 6H, compared with HA-C/EBPβ^WT^, HA-C/EBPβ^K134A^ transfection repressed the interaction of C/EBPβ and SUMO1, while transfection of HA-C/EBPβ^K134A^ or HA-C/EBPβ^ΔSUMO^ showed comparable inhibitory effect on C/EBPβ SUMOylation. Thus, these results indicated that K134 is an important site for C/EBPβ SUMOylation. To further identify whether the role of SUMO1 in cardiac hypertrophy depends on C/EBPβ K134 SUMOylation, we firstly studied the change of C/EBPβ SUMOylation in PE treated NRCMs. Though SUMO1 protein expression was increased in NRCMs treated with PE (Supplementary Figure S3B), the interaction of C/EBPβ and SUMO1 was decreased after 24 h by PE treatment (Figure 6I). Accordingly, the intracellular co-localization of SUMO1 and C/EBPβ in NRCMs were also identified by IF. As shown in Figure 6J, although PE treatment enhanced both SUMO1 and C/EBPβ level, the co-localization of SUMO1 and C/EBPβ was weakened by PE, which suggested that PE treatment restrained C/EBPβ K134 SUMOylation. Furthermore, we transfected with C/EBPβ WT or K134A mutant plasmid in NRCMs to verify whether the role of SUMO1 in cardiac hypertrophy depends on C/EBPβ K134 SUMOylation. As shown in Figure 6K, the WT C/EBPβ could significantly aggravate the ameliorative hypertrophic responses induced by SUMO1 in PE treated NRCMs, as confirmed by the changes of cell surface area (Figure 6L) and expressions of hypertrophic marker genes (Figure 6M). However, the K134A C/EBPβ only slightly aggravates the ameliorative hypertrophic responses compared with WT C/EBPβ (Figure 6K-6M). Taken together, the results above demonstrated the protective role of SUMO1 in cardiac hypertrophy depends on C/EBPβ K134 SUMOylation and its protein stability.

### The cross-talk between C/EBPβ K134 SUMOylation and PARylation participates in PARP1-induced cardiac hypertrophy

**Figure 7:**
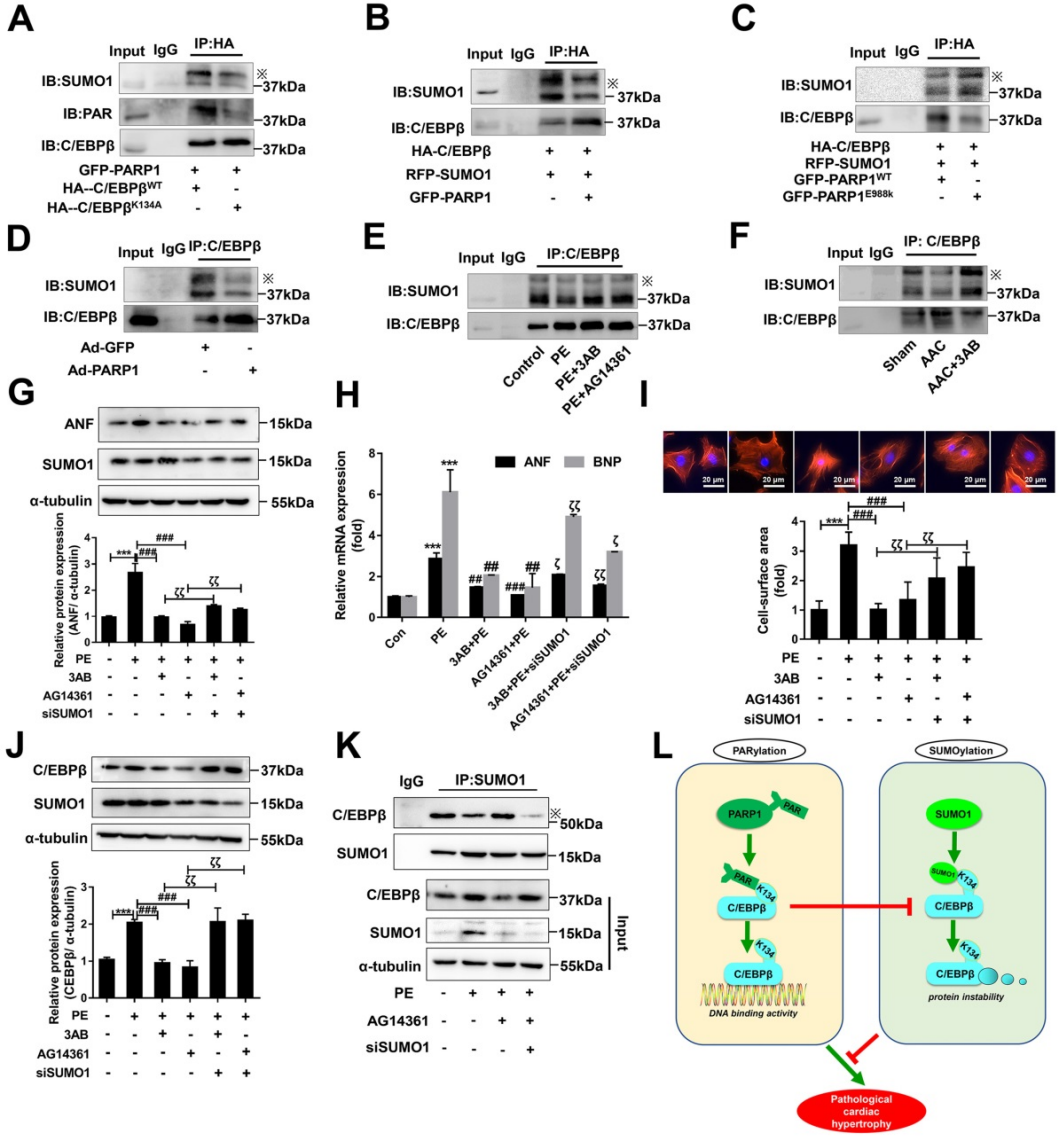
** The cross-talk between C/EBPβ K134 SUMOylation and PARylation participates in PARP1-induced cardiac hypertrophy.** (**A**) NRCMs were transfected with the expression vectors for GFP-PARP1, HA-C/EBPβ^WT^ and HA-C/EBPβ^K134A^ as indicated, immunoprecipitated with anti-HA antibody and followed by Western blot with anti-PAR, anti-SUMO1 or anti-C/EBPβ antibody. ※ indicates C/EBPβ-SUMO. n=3. (**B**,** C**) HA-C/EBPβ, RFP-SUMO1, GFP-PARP1^WT^ and GFP-PARP1^E988K^ were expressed in NRCMs as indicated. immunoprecipitated with anti-HA antibody and followed by Western blot with anti-SUMO1 or anti-C/EBPβ antibody. ※ indicates C/EBPβ-SUMO. n=3. (**D**) SD rats were submitted to intracardiac injection of adenovirus encoding PARP1 (Ad-PARP1, 10^10^ particles), the control animals received green fluorescent protein (Ad-GFP, 10^10^ particles). The rat heart tissue was immunoprecipitated with anti-C/EBPβ antibody and subsequently subjected to Western blot with anti-SUMO1 or anti-C/EBPβ antibody. ※ indicates C/EBPβ-SUMO. n=3. (**E**) NRCMs were treated with 3-aminobenzamide (3AB) (20 μmol/L) or AG14361 (1 μmol/L), followed by incubation with PE (100 μmol/L for 24 h), immunoprecipitated with anti-C/EBPβ antibody and subsequently subjected to Western blot with anti-SUMO1 or anti-C/EBPβ antibody. ※ indicates C/EBPβ-SUMO. n=3. (**F**) PARP1 inhibitor 3AB was intraperitoneally injected (20 mg/kg, twice daily) starting the week after AAC surgery for 7 weeks, the heart tissues were immunoprecipitated with anti-C/EBPβ antibody and subsequently subjected to Western blot with anti-SUMO1 or anti-C/EBPβ antibody. ※ indicates C/EBPβ-SUMO. n=3. (**G**) NRCMs were transfected with siSUMO1 or incubated with 3AB (20 μmol/L) or AG14361 (1 μmol/L) followed by incubation with PE (100 μmol/L for 24 h). The protein level of ANF was determined by western blot. ***P < 0.001 vs. Con group,^ ###^*P* < 0.001 vs. PE group, ^ζζ^*P* < 0.01 vs. PE+3AB or PE+AG14361 group, (n=3). (**H**) The mRNA levels of ANF and BNP were determined by qRT-PCR. ***P < 0.001 vs. Con group,^ ##^*P*< 0.01, ^###^*P* < 0.001 vs. PE group, ^ζ^*P* < 0.05, ^ζζ^*P* < 0.01 vs. PE+3AB or PE+AG14361 group, (n=3). (**I**) Cell surface area was measured by rhodamine staining. ***P < 0.001 vs. Con group,^ ###^*P* < 0.001 vs. PE group, ^ζζ^*P* < 0.01 vs. PE+3AB or PE+AG14361 group, (n=3). (**J**) The protein level of C/EBPβ was determined by western blot. ***P < 0.001 vs. Con group,^ ###^*P* < 0.001 vs. PE group, ^ζζ^*P* < 0.01 vs. PE+3AB or PE+AG14361 group, (n=3). (**K**) NRCMs were transfected with siSUMO1 or incubated with AG14361 (1 μmol/L) followed by incubation with PE (100 μmol/L for 24 h), immunoprecipitated with anti-SUMO1 antibody and subsequently subjected to Western blot with anti-SUMO1 or anti-C/EBPβ antibody. n=3. (**L**) Schematic model to show the posttranslational modifications of C/EBPβ and the roles they may play in pathological cardiac hypertrophy. PARP1 directly interacts with C/EBPβ and induce PARylation of C/EBPβ at K134 site in a conserved domain. The accumulation of PARylation of C/EBPβ at K134 site exhibits downregulation of C/EBPβ SUMOylation at the same site and results in upregulation of C/EBPβ protein stability. SUMO1 participates in PARP1-induced cardiac hypertrophy, which depends on SUMOylation at K134 site and protein level of C/EBPβ.

As the above results interestingly identified C/EBPβ K134 SUMOylation negatively regulate C/EBPβ protein level and improve cardiac hypertrophy, while C/EBPβ PARylation positively affect C/EBPβ protein level and aggravate cardiac hypertrophy. We speculated there may be a cross-talk between the two C/EBPβ posttranslational modifications (PTMs). To verify whether the two PTMs happened at the same site, we transfected HA-C/EBPβ WT or K134A mutant plasmid in NRCMs, it is stirring to find HA-C/EBPβ K134A transfection repressed C/EBPβ PARylation as well as SUMOylation compared with HA-C/EBPβ WT (Figure 7A), suggesting that both PARylation and SUMOylation of C/EBPβ happens at the same site (K134). We then use the gain/loss of function of C/EBPβ PARylation to explore the cross-talk between the two C/EBPβ PTMs. As shown in Figure 7B, SUMOylation of C/EBPβ was restrained when NRCMs infected with GFP-PARP1. By contrast, the level of C/EBPβ SUMOylation was rebounded when transfected with the mutant form E988K lacking PARylation activity (Figure 7C). Accordingly, in the hearts of rats injected with Ad-PARP1, which increased C/EBPβ PARylation remarkably restrained its SUMOylation (Figure 7D). Conversely, PARP1 inhibitor could reverse the suppression of C/EBPβ SUMOylation induced by PE and AAC (Figure 7E, 7F).

The results above have shown C/EBPβ PARylation inhibits its SUMOylation at K134 site, SUMO1 and PARP1 play opposite roles during the process of cardiac hypertrophy depends on C/EBPβ K134 SUMOylation and PARylation. It is worth noting whether SUMO1 was indispensable in PARP1-induced hypertrophy responses, and the crosstalk between the two PTMs in cardiac hypertrophy drew our full attention. NRCMs were transfected with SUMO1 siRNA (Supplementary Figure S1E) in the presence of PARP1 inhibitors and PE treatment. SUMO1 knockdown reversed PARP1 inhibitors-caused amelioration of cardiac hypertrophy in PE-treated NRCMs, as revealed by the increased protein level of ANF (Figure 7G), increased ANF and BNP mRNA levels (Figure 7H), and the increased cell surface area (Figure 7I). Furthermore, SUMO1 knockdown reversed PARP1 inhibitors-caused decrease of C/EBPβ protein level (Figure 7J), which could be explained by the result that SUMO1 knockdown decreased C/EBPβ SUMOylation (Figure 7K). Taken together, we have the first time demonstrated C/EBPβ PARylation inhibits C/EBPβ SUMOylation, and SUMO1 is indispensable in PARP1-induced cardiac hypertrophy because of its regulation of C/EBPβ SUMOylation and subsequent C/EBPβ protein stability.

## Discussion

Although various post-translational modifications have been extensively studied, current understanding of the crosstalk between PARylation and SUMOylation in cardiovascular disease is limited. In this study, we identified PARylation and SUMOylation of C/EBPβ at K134 site play pivotal roles in pathological cardiac hypertrophy (Figure 7L). The major novel findings of the present study include: (1) PARP1 directly interacts with C/EBPβ and induce PARylation of C/EBPβ at K134 site in a conserved domain; (2) The accumulation of PARylation of C/EBPβ at K134 site exhibits downregulation of C/EBPβ SUMOylation at the same site and results in upregulation of C/EBPβ protein stability; (3) SUMO1 participates in PARP1-induced cardiac hypertrophy, which depends on SUMOylation at K134 site and protein level of C/EBPβ.

PARP1 has been regarded as a promising target for pathological cardiac hypertrophy 33, 34. PARP1 activity is enhanced in the progression of cardiac hypertrophy, while silencing or inhibition of PARP1 alleviates cardiac hypertrophy. Overexpression of PARP1 exacerbates cardiomyocyte hypertrophy, whereas the hypertrophic responses are prevented by C/EBPβ knockdown. PARP1 transfers poly-ADP-ribose (PAR) groups from donor NAD^+^ molecules onto their target substrates, this PTM called poly(ADP-ribosyl)ation (PARylation) 16. Here, we found that PARP1 directly PARylates C/EBPβ in cardiomyocytes and heart tissues. The protein level of C/EBPβ is upregulated by PARP1 overexpression, while is suppressed by PARP1 knockdown or inhibition. The present work demonstrates the first time that the aggravating role of PARP1 in cardiac hypertrophy is partially attribute to its upregulation of C/EBPβ protein.

In cardiac hypertrophy model, the mRNA level of C/EBPβ is unaltered, but its protein level and DNA binding activity are upregulated, which may be due to the increase of protein stability or inhibition of protein degradation. The stability or degradation of C/EBPβ protein is mainly affected by C/EBPβ SUMOylation and subsequent ubiquitination 37. In this study, we found C/EBPβ PARylation is augmented, while its SUMOylation is reduced in PE or AAC-induced cardiac hypertrophy. Similar results were observed in PARP1-induced cardiac hypertrophy. Our data suggest that C/EBPβ PARylation and SUMOylation, may play important roles for regulating its protein level and in the process of cardiac hypertrophy.

The role of SUMO1 in cardiovascular diseases has recently been studied, SERCA2a is SUMOylated (at lysine 480 and 585 site) by SUMO1 and this SUMOylation is essential for maintaining ATPase activity and SERCA2a stability, which could rescue pressure overload-induced heart failure 40, miR-146a is a novel regulator or upstream of the SUMOylation machinery in the heart 41. In line with the protective role of SUMO1 reported in cardiac dysfunction, we further uncovered the underlying mechanisms of SUMO1 in the process of cardiac hypertrophy by the following findings: (1) SUMO1 regulates C/EBPβ protein stability; (2) The protective role of SUMO1 in cardiac hypertrophy depends on SUMOylation of C/EBPβ at K134 site; (3) Depletion of PARP1 cannot inhibit cardiac hypertrophy without SUMO1 expression in cardiomyocytes.

Previous studies have reported that on the RD motif (LKAEP) in rat C/EBPβ, 134 lysine residue (K134) is considered the likely site for attachment of SUMO1 15, 37, 38. C/EBPβ interacts with PIAS1 (a SUMO E3 ligase) and is SUMOylated at Lys133 (K134 in rat) site, inducing ubiquitination and degradation of C/EBPβ protein 39. We found that PARP1-mediated PARylation of C/EBPβ inhibits its SUMOylation induced by SUMO1 at K134 site in cardiomyocytes. C/EBPβ single mutant with alanine substitution at K134 (mutant K134A) or truncation of conserved motif LKAEP (mutant ΔSUMO) inhibits PARylation and SUMOylation of C/EBPβ in cardiomyocytes, suggesting that K134 site is a common site for these two post-translational modifications. The increased PARP1 catalytic activity may induce C/EBPβ K134 site be largely occupied by PAR chain, blocking SUMO1-mediated K134 SUMOylation, subsequently enhances its protein stability.

According to our work, it is demonstrated the accumulation of PARylation of C/EBPβ at K134 site exhibits downregulation of C/EBPβ SUMOylation at the same site. However, the definite spatial-temporal information of interactions among three proteins (PARP1, SUMO1 and C/EBPβ) remains to be investigated under normal condition. SUMOylation is catalyzed by SUMO-specific ligases while could be reversed by Sentrin/SUMO-specific proteases (SENPs), which is a dynamic process. In this study, whether the interaction or modification happens after C/EBPβ deSUMOylation in nucleus, and whether other deSUMOylation enzymes are involved in C/EBPβ PARylation are not fully understood. Furthermore, although it is reported SUMOylation of C/EBPβ significantly increased its ubiquitination and instability 37, the underlying mechanism remains largely unknown. The future study would focus on the screening for C/EBPβ SUMOylation-associated ubiquitin ligase that mediates C/EBPβ protein degradation. Finally, cardiac-specific C/EBPβ mutant K134A or mutant ΔSUMO mice would unambiguously address questions about the roles of C/EBPβ and PARP1 in the process of cardiac hypertrophy. The previous study has yielded evidence of PARP1 activation in myocardial samples from patients with heart failure 42. The SUMO1 mRNA level was demonstrated decreased in human hearts of heart failure patients 41. Depending on this study that the opposing role of C/EBPβ PARylation (aggravating) and SUMOylation (alleviative) in cardiac hypertrophy, it is clear that further experiments are required to clarify the relationship between the PTMs of C/EBPβ in failing human heart samples.

In summary, our study reveals novel mechanisms for the cross-talk between C/EBPβ PARylation and SUMOylation at same site (K134) in the process of cardiac hypertrophy. Given that emerging and clear evidence has shown PAPR1 inhibitors could improve the life quality for patients suffering cancer and cardiovascular diseases 43-45, multi-target therapeutic methods inhibiting PARP1 and activating C/EBPβ SUMOylation would be potential for treating pathological cardiac hypertrophy.

## Supplementary Material

Supplementary figures and tables.

## Abbreviations

AAC: abdominal aortic constriction
ANF: atrial natriuretic factor
AngII: angiotensin II
BNP: brain natriuretic polypeptide
C/EBP: CCAAT/enhancer-binding protein
HE: hematoxylin-eosin
IF: immunofluorescence
IHC: immunohistochemical
NAD: nicotinamide adenine dinucleotide
NRCMs: neonatal rat cardiomyocytes
PARP1: poly(ADP-ribose) polymerase-1
PARylation: poly(ADP-ribosyl)ation
PARylated: poly(ADP-ribosyl)ated
PAR: polymers of ADP-ribose
PE: phenylephrine
SUMO: small ubiquitin-like modifier
SUMOylation: SUMO modification
3AB: 3-aminobenzamide
