# Supplementary material for: The cross-talk between PARylation and SUMOylation in C/EBPβ at K134 site participates in pathological cardiac hypertrophy
Source: Int J Biol Sci. 2022 Jan 1;18(2):783–99. doi: 10.7150/ijbs.65211 (PMC8741850; doi:10.7150/ijbs.65211)
Supplement: Supplementary file 1 — Supplementary figures and tables. [file ijbsv18p0783s1.pdf]

SUPPLEMENTARY MATERIAL

The cross-talk between PARylation and SUMOylation in C/EBPβ at K134 site participates in pathological cardiac hypertrophy

Luping Wang[1,2,#], Panxia Wang[1,#], Suowen Xu[3], Zhuoming Li[1], Dayue Darrel Duan[4], Jiantao Ye[1], Jingyan Li[1], Yanqing Ding[1], Wenqing Zhang[2], Jing Lu[1,*] ,Peiqing Liu[1,5,6,*]

[1]Department of Pharmacology and Toxicology, School of Pharmaceutical Sciences, Sun Yat-Sen University, Guangdong, China;

[2]Laboratory of Hematopathology & Drug Discovery, School of Medicine, South China University of Technology, Guangdong, China;

[3]Department of Endocrinology, The First Affiliated Hospital of USTC, Division of Life Sciences and Medicine, University of Science and Technology of China, Anhui, China;

[4]Center for Phenomics of Traditional Chinese Medicine/the Affiliated Hospital of Traditional Chinese Medicine, Southwest Medical University, Sichuan, China;

[5]National and Local United Engineering Lab of Druggability and New Drugs Evaluation, School of Pharmaceutical Sciences, Sun Yat-Sen University, Guangdong, China;

[6]School of Pharmacy, Jinan University, 601 Huangpu Avenue West, Guangdong, China.

* Correspondence: School of Pharmaceutical Sciences, Sun Yat-sen University (Higher Education Mega Center), 132# East Wai-huan Road, Guangzhou 510006, Guangdong, China. Tel.: +86 20 39943116; fax: +86 20 39943026.

E-mail addresses: Jing Lu (lujing0504@126.com); Peiqing Liu (liupq@mail.sysu.edu.cn)

[#]These authors contributed equally: Luping Wang, Panxia Wang

Supplementary Figures and Figure Legends

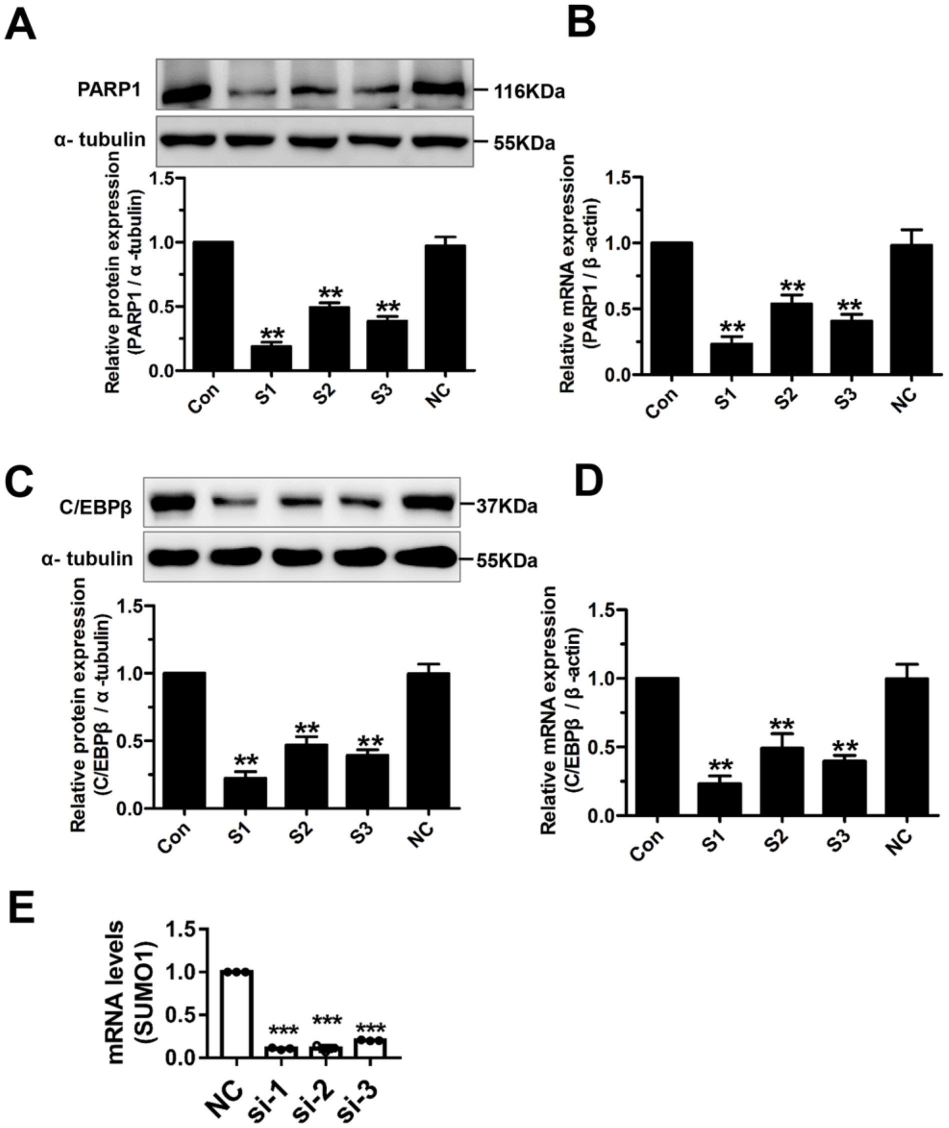

Figure S1. qPCR and Western blot were conducted to confirm the silencing efficiency of siRNAs. (**A** and **B**) The silencing efficiency of three different siRNA against PARP1 (S1, S2 and S3) was tested. (**C** and **D**) The silencing efficiency of three different siRNA against C/EBPβ (S1, S2 and S3) was tested. (**E**) The silencing efficiency of three different siRNA against SUMO1 (si-1, si-2 and si-3) was tested. $^{**}P < 0.01$ *vs.* the NC group, $^{***}P < 0.001$ *vs.* the NC group. n=5.

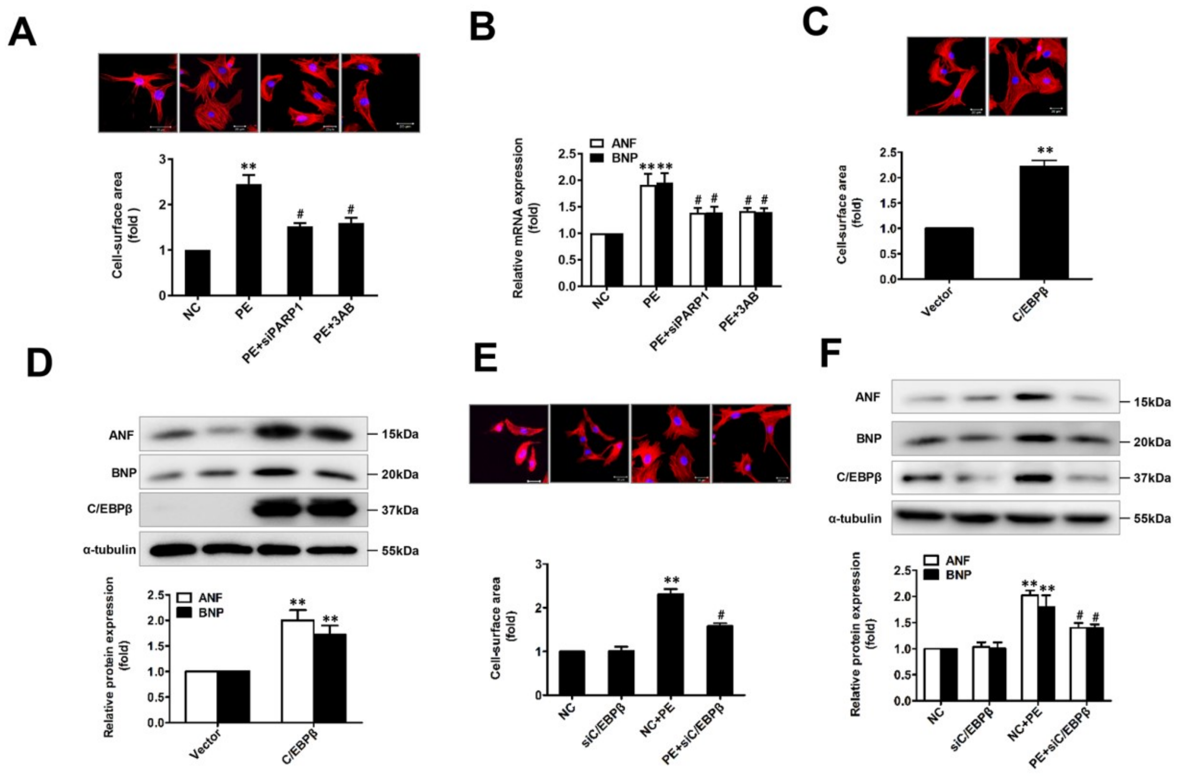

Figure S2. PARP1 and C/EBPβ participate in cardiac hypertrophy. NRCMs were transfected with siPARP1 or incubated with 3-aminobenzamide (3AB) (20 μmol/L) followed by incubation with phenylephrine (PE) (100 μmol/L for 24 h). (**A**) the cell surface area was observed using rhodamine-phalloidin staining, and the mRNA levels of atrial natriuretic factor (ANF) and brain natriuretic polypeptide (BNP) (**B**) were determined by qRT-PCR. NRCMs were transfected with HA-C/EBPβ plasmid and the hypertrophic responses were determined by measuring cell surface area (**C**) and the protein levels of ANF and BNP (**D**). NRCMs were transfected with siC/EBPβ followed by incubation with PE (100 μmol/L for 24 h), the reversed hypertrophic responses were demonstrated by cell surface area (**E**) and the protein levels of ANF and BNP (**F**). *$P < 0.05$ vs. the Control group, **$P < 0.01$ vs. the Control group, $^{\#}P < 0.05$ vs. the PE treatment group. n=3.

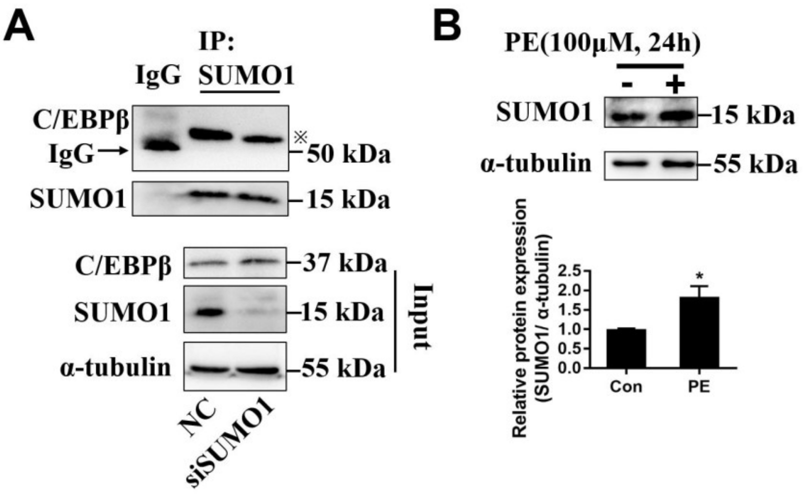

Figure S3. **A**, NRCMs were transfected with SUMO1 siRNA, immunoprecipitated with anti-SUMO1 antibody and subsequently subjected to immunoblotting analysis. ※ indicates C/EBPβ-SUMO. **B**, Neonatal rat cardiomyocytes (NRCMs) were treated with PE (100 μmol/L for 24 h), the protein level of SUMO1 was determined by western blotting analysis. *n*=3.

```
Sequence ID: lcl|Query_40385  Length: 1014  Number of Matches: 1

Range 1: 1 to 1014 Graphics                              ▼Next Match ▲Previous Match
Score            Expect Method                        Identities       Positives         Gaps
2085 bits(5403) 0.0    Compositional matrix adjust. 1013/1014(99%) 1014/1014(100%) 0/1014(0%)

Query  1     MAEATERLYRVEYAKSGRASCKKCSESIPKDSLRMAIMVQSPMFDGKVPHWYHFSCFWKV  60
             MAEATERLYRVEYAKSGRASCKKCSESIPKDSLRMAIMVQSPMFDGKVPHWYHFSCFWKV
Sbjct  1     MAEATERLYRVEYAKSGRASCKKCSESIPKDSLRMAIMVQSPMFDGKVPHWYHFSCFWKV  60

Query  61    GHSIRQPDTEVDGFSELRWDDQQKVKKTAEAGGVAGKGQHGGGGKAEKTLGDFAAEYAKS  120
             GHSIRQPDTEVDGFSELRWDDQQKVKKTAEAGGVAGKGQHGGGGKAEKTLGDFAAEYAKS
Sbjct  61    GHSIRQPDTEVDGFSELRWDDQQKVKKTAEAGGVAGKGQHGGGGKAEKTLGDFAAEYAKS  120

Query  121   NRSTCKGCMEKIEKGQMRLSKKMLDPEKPQLGMIDRWYHPTCFVKNRDELGFRPEYSASQ  180
             NRSTCKGCMEKIEKGQMRLSKKMLDPEKPQLGMIDRWYHPTCFVKNRDELGFRPEYSASQ
Sbjct  121   NRSTCKGCMEKIEKGQMRLSKKMLDPEKPQLGMIDRWYHPTCFVKNRDELGFRPEYSASQ  180

Query  181   LKGFSLLSAEDKEALKKQLPAVKSEGKRKCDEVDGIDEVAKKKSKKGKDKESSKLEKALK  240
             LKGFSLLSAEDKEALKKQLPAVKSEGKRKCDEVDGIDEVAKKKSKKGKDKESSKLEKALK
Sbjct  181   LKGFSLLSAEDKEALKKQLPAVKSEGKRKCDEVDGIDEVAKKKSKKGKDKESSKLEKALK  240

Query  241   AQNELVWNIKDELKKACSTNDLKELLIFNQQQVPSGESAILDRVADGMAFGALLPCKECS  300
             AQNELVWNIKDELKKACSTNDLKELLIFNQQQVPSGESAILDRVADGMAFGALLPCKECS
Sbjct  241   AQNELVWNIKDELKKACSTNDLKELLIFNQQQVPSGESAILDRVADGMAFGALLPCKECS  300

Query  301   GQLVFKSDAYYCTGDVTAWTKCMVKTQNPSRKEWVTPKEFREISYLKKLKIKKQDRLFPP  360
             GQLVFKSDAYYCTGDVTAWTKCMVKTQNPSRKEWVTPKEFREISYLKKLKIKKQDRLFPP
Sbjct  301   GQLVFKSDAYYCTGDVTAWTKCMVKTQNPSRKEWVTPKEFREISYLKKLKIKKQDRLFPP  360

Query  361   ESSAPAPPAPPVSITSAPTAVNSSAPADKPLSNMKILTLGKLSQNKDEAKAMIEKLGGKL  420
             ESSAPAPPAPPVSITSAPTAVNSSAPADKPLSNMKILTLGKLSQNKDEAKAMIEKLGGKL
Sbjct  361   ESSAPAPPAPPVSITSAPTAVNSSAPADKPLSNMKILTLGKLSQNKDEAKAMIEKLGGKL  420

Query  421   TGSANKASLCISTKKEVEKMSKKMEEVKAANVRVVCEDFLQDVSASAKSLQELLSAHSLS  480
             TGSANKASLCISTKKEVEKMSKKMEEVKAANVRVVCEDFLQDVSASAKSLQELLSAHSLS
Sbjct  421   TGSANKASLCISTKKEVEKMSKKMEEVKAANVRVVCEDFLQDVSASAKSLQELLSAHSLS  480

Query  481   SWGAEVKVEPGEVVVPKGKSAAPSKKSKGAVKEEGVNKSEKRMKLTLKGGAAVDPDSGLE  540
             SWGAEVKVEPGEVVVPKGKSAAPSKKSKGAVKEEGVNKSEKRMKLTLKGGAAVDPDSGLE
Sbjct  481   SWGAEVKVEPGEVVVPKGKSAAPSKKSKGAVKEEGVNKSEKRMKLTLKGGAAVDPDSGLE  540

Query  541   HSAHVLEKGGKVFSATLGLVDIVKGTNSYYKLQLLESDKESRYWIFRSWGRVGTVIGSNK  600
             HSAHVLEKGGKVFSATLGLVDIVKGTNSYYKLQLLESDKESRYWIFRSWGRVGTVIGSNK
Sbjct  541   HSAHVLEKGGKVFSATLGLVDIVKGTNSYYKLQLLESDKESRYWIFRSWGRVGTVIGSNK  600

Query  601   LEQMPSKEDAVEHFMKLYEEKTGNAWHSKNFTKYPKKFYPLEIDYGQDEEAVKKLAVKPG  660
             LEQMPSKEDAVEHFMKLYEEKTGNAWHSKNFTKYPKKFYPLEIDYGQDEEAVKKLAVKPG
Sbjct  601   LEQMPSKEDAVEHFMKLYEEKTGNAWHSKNFTKYPKKFYPLEIDYGQDEEAVKKLAVKPG  660

Query  661   TKSKLPKPVQELVGMIFDVESMKKALVEYEIDLQKMPLGKLSRRQIQAAYSILSEVQQAV  720
             TKSKLPKPVQELVGMIFDVESMKKALVEYEIDLQKMPLGKLSRRQIQAAYSILSEVQQAV
Sbjct  661   TKSKLPKPVQELVGMIFDVESMKKALVEYEIDLQKMPLGKLSRRQIQAAYSILSEVQQAV  720

Query  721   SQGSSESQILDLSNRFYTLIPHDFGMKKPPLLNNTDSVQAKVEMLDNLLDIEVAYSLLRG  780
             SQGSSESQILDLSNRFYTLIPHDFGMKKPPLLNNTDSVQAKVEMLDNLLDIEVAYSLLRG
Sbjct  721   SQGSSESQILDLSNRFYTLIPHDFGMKKPPLLNNTDSVQAKVEMLDNLLDIEVAYSLLRG  780

Query  781   GSDDSSKDPIDVNYEKLKTDIKVVDRDSEEAEVIRKYVKNTHATTHNAYDLEVIDIFKIE  840
             GSDDSSKDPIDVNYEKLKTDIKVVDRDSEEAEVIRKYVKNTHATTHNAYDLEVIDIFKIE
Sbjct  781   GSDDSSKDPIDVNYEKLKTDIKVVDRDSEEAEVIRKYVKNTHATTHNAYDLEVIDIFKIE  840

Query  841   REGESQRYKPFRQLHNRRLLWHGSRTTNFAGILSQGLRIAPPEAPVTGYMFGKGIYFADM  900
             REGESQRYKPFRQLHNRRLLWHGSRTTNFAGILSQGLRIAPPEAPVTGYMFGKGIYFADM
Sbjct  841   REGESQRYKPFRQLHNRRLLWHGSRTTNFAGILSQGLRIAPPEAPVTGYMFGKGIYFADM  900

Query  901   VSKSANYCHTSQGDPIGLILLGEVALGNMYELKHASHISKLPKGKHSVKGLGKTAPDPSA  960
             VSKSANYCHTSQGDPIGLILLGEVALGNMYELKHASHISKLPKGKHSVKGLGKTAPDPSA
Sbjct  901   VSKSANYCHTSQGDPIGLILLGEVALGNMYELKHASHISKLPKGKHSVKGLGKTAPDPSA  960

Query  961   SITLDGVEVPLGTGIPSGVNDTCLLYNEYIVYDIAQVNLKYLLKLKFNFKTSLW  1014
             SITLDGVEVPLGTGIPSGVNDTCLLYN+YIVYDIAQVNLKYLLKLKFNFKTSLW
Sbjct  961   SITLDGVEVPLGTGIPSGVNDTCLLYNKYIVYDIAQVNLKYLLKLKFNFKTSLW  1014
```

Figure S4. The protein sequences of PARP1 and its mutant (E988K) were aligned using Basic Local Alignment Search Tool (BLAST). We produced a mutant form of PARP1, E988K, which lacks catalytic activity. Glutamic acid (E) in the 988 site of PARP1 was replaced by lysine residue (K), and its protein sequences were aligned to wild type PARP1.

Sequence ID: Query_186913 Length: 297 Number of Matches: 1

Range 1: 1 to 297 Graphics ▼ Next Match ▲ Previous Match

Score	Expect	Method	Identities	Positives	Gaps
580 bits(1496)	0.0	Compositional matrix adjust.	296/297(99%)	296/297(99%)	0/297(0%)

```
Query  1    MHRLLAWDAACLPPPPAAFRPMEVANFYYEPDCLAYGAKAARAAPRAPAAEPAIGEHERA  60
            MHRLLAWDAACLPPPPAAFRPMEVANFYYEPDCLAYGAKAARAAPRAPAAEPAIGEHERA
Sbjct  1    MHRLLAWDAACLPPPPAAFRPMEVANFYYEPDCLAYGAKAARAAPRAPAAEPAIGEHERA  60

Query  61   IDFSPYLEPLAPAAADFAAPAPAHHDFLSDLFADDYGAKPSKKPSDYGYVSLGRAGAKAA  120
            IDFSPYLEPLAPAAADFAAPAPAHHDFLSDLFADDYGAKPSKKPSDYGYVSLGRAGAKAA
Sbjct  61   IDFSPYLEPLAPAAADFAAPAPAHHDFLSDLFADDYGAKPSKKPSDYGYVSLGRAGAKAA  120

Query  121  PPACFPPPPPAALKAEPGFEPADCKRADDAPAMAAGFPFALRAYLGYQATPSGSSGSLST  180
            PPACFPPPPPAAL AEPGFEPADCKRADDAPAMAAGFPFALRAYLGYQATPSGSSGSLST
Sbjct  121  PPACFPPPPPAALAAEPGFEPADCKRADDAPAMAAGFPFALRAYLGYQATPSGSSGSLST  180

Query  181  SSSSSPPGTPSPADAKAAPAACFAGPPAAPAKAKAKKAVDKLSDEYKMRRERNNIAVRKS  240
            SSSSSPPGTPSPADAKAAPAACFAGPPAAPAKAKAKKAVDKLSDEYKMRRERNNIAVRKS
Sbjct  181  SSSSSPPGTPSPADAKAAPAACFAGPPAAPAKAKAKKAVDKLSDEYKMRRERNNIAVRKS  240

Query  241  RDKAKMRNLETQHKVLELTAENERLQKKVEQLSRELSTLRNLFKQLPEPLLASAGHC  297
            RDKAKMRNLETQHKVLELTAENERLQKKVEQLSRELSTLRNLFKQLPEPLLASAGHC
Sbjct  241  RDKAKMRNLETQHKVLELTAENERLQKKVEQLSRELSTLRNLFKQLPEPLLASAGHC  297
```

Figure S5. The protein sequences of C/EBPβ and its mutant (K134A) were aligned using Basic Local Alignment Search Tool (BLAST). We produced a mutant form of C/EBPβ, Lysine acid (K) at the 134 site of C/EBPβ was replaced by Arginine residue (A), and its protein sequences were aligned to wild type C/EBPβ.

Sequence ID: Query_223603 Length: 292 Number of Matches: 1

Range 1: 1 to 292 Graphics ▼ Next Match ▲ Previous Match

Score	Expect	Method	Identities	Positives	Gaps
568 bits(1465)	0.0	Compositional matrix adjust.	292/297(98%)	292/297(98%)	5/297(1%)

```
Query  1    MHRLLAWDAACLPPPPAAFRPMEVANFYYEPDCLAYGAKAARAAPRAPAAEPAIGEHERA  60
            MHRLLAWDAACLPPPPAAFRPMEVANFYYEPDCLAYGAKAARAAPRAPAAEPAIGEHERA
Sbjct  1    MHRLLAWDAACLPPPPAAFRPMEVANFYYEPDCLAYGAKAARAAPRAPAAEPAIGEHERA  60

Query  61   IDFSPYLEPLAPAAADFAAPAPAHHDFLSDLFADDYGAKPSKKPSDYGYVSLGRAGAKAA  120
            IDFSPYLEPLAPAAADFAAPAPAHHDFLSDLFADDYGAKPSKKPSDYGYVSLGRAGAKAA
Sbjct  61   IDFSPYLEPLAPAAADFAAPAPAHHDFLSDLFADDYGAKPSKKPSDYGYVSLGRAGAKAA  120

Query  121  PPACFPPPPPAALKAEPGFEPADCKRADDAPAMAAGFPFALRAYLGYQATPSGSSGSLST  180
            PPACFPPPPPAA     GFEPADCKRADDAPAMAAGFPFALRAYLGYQATPSGSSGSLST
Sbjct  121  PPACFPPPPPAA-----GFEPADCKRADDAPAMAAGFPFALRAYLGYQATPSGSSGSLST  175

Query  181  SSSSSPPGTPSPADAKAAPAACFAGPPAAPAKAKAKKAVDKLSDEYKMRRERNNIAVRKS  240
            SSSSSPPGTPSPADAKAAPAACFAGPPAAPAKAKAKKAVDKLSDEYKMRRERNNIAVRKS
Sbjct  176  SSSSSPPGTPSPADAKAAPAACFAGPPAAPAKAKAKKAVDKLSDEYKMRRERNNIAVRKS  235

Query  241  RDKAKMRNLETQHKVLELTAENERLQKKVEQLSRELSTLRNLFKQLPEPLLASAGHC  297
            RDKAKMRNLETQHKVLELTAENERLQKKVEQLSRELSTLRNLFKQLPEPLLASAGHC
Sbjct  236  RDKAKMRNLETQHKVLELTAENERLQKKVEQLSRELSTLRNLFKQLPEPLLASAGHC  292
```

Figure S6. The protein sequences of C/EBPβ and its truncated mutant (ΔSUMO) were aligned using Basic Local Alignment Search Tool (BLAST). The conserved motif (LKAEP) was truncated, which lacks the sites for poly(ADP-ribosyl)ation, and its protein sequences were aligned to wild type C/EBPβ.

Supplementary Tables

Table S1. Rat-specific primer sequences for quantitative reverse transcription polymerase chain reaction (qRT-PCR)

Target gene	Sequences
ANF	Forward: 5'-GGAAGTCAACCCGTCTCA-3'
	Reverse: 5'-AGCCCTCAGTTTGCTTTT-3'
BNP	Forward: 5'-TTTGGGCAGAAGATAGACCG-3'
	Reverse: 5'-AGAAGAGCCGCAGGCAGAG-3'
C/EBPβ	Forward:5'-AAGATGCGCAACCTGGAGAC-3'
	Reverse:5'-CCTTCTTCTGCAGCCGCTC-3'
SUMO1	Forward:5'-TCCAAAAGTGGCTTCACTGGA -3'
	Reverse:5'-AGCAGTGTCTGTTGCGTACA-3'
β-actin	Forward: 5'-TCGTGCGTGACATTAAAGAG-3'
	Reverse: 5'-ATTGCCGATAGTGATGACCT-3'

Table S2. Sequences of siRNAs for C/EBPβ, PARP1 and SUMO1

siRNA	Sequences
C/EBPβ-rat-01	Sense: 5'-ACCUCUUCGCCGACGACUATT-3'
	Antisense:5'-UAGUCGUCGGCGAAGAGGUTT-3'
C/EBPβ-rat-02	Sense:5'-AGCUGAGCGACGAGUACAATT-3'
	Antisense: 5'-UUGUACUCGUCGCUCAGCUTT-3'
C/EBPβ-rat-03	Sense:5'-CCAUGGAAGUGGCCAACUUTT-3'
	Antisense:5'-AAGUUGGCCACUUCCAUGGTT-3'
PARP1-rat-01	Sense: 5'-GGGACUAACUCCUAUUACATT-3'
	Antisense:5'-UGUAAUAGGAGUUAGUCCCTT-3'
PARP1-rat-02	Sense: 5'-CCAUGUUCGAUGGAAAAGUTT -3'
	Antisense:5'-ACUUUUCCAUCGAACAUGGTT -3'
PARP1-rat-03	Sense: 5'-CCUACCUCAAGAAAUUAAATT -3'
	Antisense:5'-UUUAAUUUCUUGAGGUAGGTT-3'

SUMO1-rat-01	Sense: 5'-GCAGUGAGAUCCAUUUCAATT-3'
	Antisense:5'-UUGAAAUGGAUCUCACUGCTT-3'
SUMO1-rat-02	Sense: 5'-GGAAGAAGACGUGAUUGAATT-3'
	Antisense:5'-UUCAAUCACGUCUUCUUCCTT-3'
SUMO1-rat-03	Sense: 5'- GACAGGGAGUUCCAAUGAATT-3'
	Antisense:5'-UUCAUUGGAACUCCCUGUCTT-3'
NC	Sense:5'- UUCUCCGAACGUGUCACGUTT-3'
	Antisense:5'- ACGUGACACGUUCGGAGAATT-3'

Table S3. Echocardiographic parameters of rats submitted to intramyocardial injection of adenovirus encoding PARP1 and ALA intraperitoneal injection

Group	Ad-GFP	Ad-PARP1	Ad-PARP1+ALA
LVAWd (mm)	1.62±0.04	2.32±0.09*	2.08±0.07
LVAWs(mm)	2.05±0.19	3.12±0.12*	2.89±0.04
LVIDd(mm)	7.62±0.29	7.36±0.47*	7.78±0.08#
LVIDs(mm)	4.90±0.13	4.32±0.13*	4.55±0.11
LVPWd(mm)	2.16±0.08	2.32±0.16*	2.21±0.04#
LVPWs(mm)	3.08±0.09	3.49±0.26*	3.28±0.02#
EF%	62.38±4.9	70.32±2.91*	64.92±2.25#
FS%	34.95±2.8	40.59±1.65*	38.46±1.02#
SV(μL)	219.25±12.8	274.36±14.2*	252.27±13.2#
LVEDV(μL)	358.05±18.3	390.25±17.8*	382.36±16.2
LVSV(μL)	165.35±10.8	175.26±9.6	168.26±10.3

Data were presented as mean ± SD. **P*<0.05 *vs*. Ad-GFP group, #P < 0.05 *vs*. Ad-PARP1 group, *n*=8.

Table S4. Echocardiographic parameters of rats submitted to abdominal aortic constriction (AAC) surgery and 3AB intraperitoneal injection

**Group**	**Sham**	**AAC**	**AAC+3AB**
IVS-d (mm)	1.64±0.06	2.25±0.06*	1.82±0.04#
IVS-s (mm)	2.26±0.10	3.16±0.08*	2.69±0.07#
LVID-d (mm)	8.01±0.09	7.32±0.07*	7.67±0.09#
LVID-s (mm)	5.34±0.21	4.21±0.14*	4.97±0.11#
LVPW-d (mm)	1.82±0.08	2.43±0.09*	2.09±0.04#
LVPW-s (mm)	2.64±0.06	3.34±0.09*	2.98±0.04#
EF%	61.80±1.60	71.63±1.84*	64.92±2.29#
FS%	36.00±1.50	45.48±1.10*	40.46±1.04#
SV(μL)	221.25±11.6	278.36±14.1*	248.22±11.2#
LVSV(μL)	348.05±15.7	387.24±18.2*	378.36±14.3
LVEDV(μL)	159.35±11.4	165.26±9.6	164.26±10.3

Data are presented as mean ± SD. *P<0.05 *vs*. the sham group, #P < 0.05 *vs*. the AAC group, n = 8.
